# Learning from Ethiopia’s success in reducing maternal and neonatal mortality through a health systems lens

**DOI:** 10.1136/bmjgh-2023-011911

**Published:** 2024-05-06

**Authors:** Dessalegn Y. Melesse, Ashenif Tadele, Shegaw Mulu, Neil Spicer, Tefera Tadelle, Yohannes D Wado, Mulugeta Gajaa, Asrat Arja, Cauane Blumenberg, Tewabe Manaye, Geremew Gonfa, Elsabe du Plessis, Elisabeth Hamilton, Awoke Mihretu, Abdurehman Usamael, Magdelawit Mengesha, Solomon Kassahun Gelaw, Aschale Worku, Mirkuzie Woldie, Biruk Abate, Theodros Getachew, Naod Wondirad, Meseret Zelalem, Getachew Tollera, Ties Boerma

**Affiliations:** 1 Countdown to 2030 for Women's, Children's and Adolescents' Health, Institute for Global Public Health, Department of Community Health Sciences, University of Manitoba, Winnipeg, Manitoba, Canada; 2 Department of Epidemiology and Biostatistics, School of Public Health, Bahir Dar University, Bahir Dar, Ethiopia; 3 Health System and Reproductive Health Research Directorate, Ethiopian Public Health Institute, Addis Ababa, Ethiopia; 4 Maternal and Child Health Directorate, Federal Ministry of Health, Addis Ababa, Ethiopia; 5 Department of Global Health and Development, London School of Hygiene & Tropical Medicine, London, UK; 6 African Population and Health Research Center, Nairobi, Kenya; 7 National Data Management Center for Health, Ethiopian Public Health Institute, Addis Ababa, Ethiopia; 8 International Center for Equity in Health, Federal University of Pelotas, Pelotas, Brazil; 9 causale consultoria, Pelotas, Brazil; 10 Policy, Planning, Monitoring & Evaluation Directorate, Ethiopia Ministry of Health, Addis Ababa, Ethiopia; 11 Maternal, Child and Adolescent Health Lead Executive, Federal Ministry of Health, Addis Ababa, Ethiopia

**Keywords:** Health systems, Maternal health, Public Health, Health policy, Child health

## Abstract

**Background:**

This study aimed to enhance insights into the key characteristics of maternal and neonatal mortality declines in Ethiopia, conducted as part of a seven-country study on Maternal and Newborn Health (MNH) Exemplars.

**Methods:**

We synthesised key indicators for 2000, 2010 and 2020 and contextualised those with typical country values in a global five-phase model for a maternal, stillbirth and neonatal mortality transition. We reviewed health system changes relevant to MNH over the period 2000–2020, focusing on governance, financing, workforce and infrastructure, and assessed trends in mortality, service coverage and systems by region. We analysed data from five national surveys, health facility assessments, global estimates and government databases and reports on health policies, infrastructure and workforce.

**Results:**

Ethiopia progressed from the highest mortality phase to the third phase, accompanied by typical changes in terms of fertility decline and health system strengthening, especially health infrastructure and workforce. For health coverage and financing indicators, Ethiopia progressed but remained lower than typical in the transition model. Maternal and neonatal mortality declines and intervention coverage increases were greater after 2010 than during 2000–2010. Similar patterns were observed in most regions of Ethiopia, though regional gaps persisted for many indicators. Ethiopia’s progress is characterised by a well-coordinated and government-led system prioritising first maternal and later neonatal health, resulting major increases in access to services by improving infrastructure and workforce from 2008, combined with widespread community actions to generate service demand.

**Conclusion:**

Ethiopia has achieved one of the fastest declines in mortality in sub-Saharan Africa, with major intervention coverage increases, especially from 2010. Starting from a weak health infrastructure and low coverage, Ethiopia’s comprehensive approach provides valuable lessons for other low-income countries. Major increases towards universal coverage of interventions, including emergency care, are critical to further reduce mortality and advance the mortality transition.

**WHAT IS ALREADY KNOWN ON THIS TOPIC**

- Maternal and neonatal mortality in Ethiopia declined by 72% and 44%, respectively, during the first two decades of the 21st century, faster than in most other countries in sub-Saharan Africa.
- Ethiopia’s overall and child health progress has been attributed to the government’s leadership, the Health Extension Programme and its multisectoral approach to development.

**WHAT THIS STUDY ADDS**

- Benchmarking Ethiopia’s progress in a mortality transition model shows that many of the changes were typical for a country moving rapidly out of the highest mortality phase, characterised by strong fertility decline and increased access to and utilisation of services. However, coverage of maternal and newborn health (MNH) interventions, such as institutional delivery and caesarean section were lower than typical coverage observed in countries in the same phase in the maternal and neonatal mortality transition.
- Ethiopia’s progress in coverage for MNH indicators was characterised by its major expansion in health infrastructure and health workforce, supported by multiple initiatives such as task shifting to increase surgical capacity, increasing availability of ambulances, maternity waiting homes and generating demand through community volunteers, accompanied by prorural development approaches, such as expanding road networks.
- Ethiopia was successful in bringing about progress in all its regions, with its large population regions of Oromia, Amhara and Southen Nations, Nationalities and Poeple’s Region (SNNPR) driving national progress, but large differences between regions persist, and regions with high numbers of pastoralists are lagging.

**HOW THIS STUDY MIGHT AFFECT RESEARCH, PRACTICE OR POLICY**

- Ethiopia’s experience provides valuable lessons for other countries with higher mortality and limited resources, including its comprehensive health system approach to advance MNH programmes and the first steps towards a learning health system approach, characterised by good health governance and use of data and reviews to monitor progress and performance of the health system.
- Survival gains can only be sustained and expanded if coverage and quality of all MNH interventions continue to increase, especially in the more populous and pastoralist regions in Ethiopia, and special attention is paid to increasing deliveries in health facilities that can provide emergency obstetric and newborn care.

## Introduction

 Before 2000, Ethiopia had rudimentary rural health infrastructure, poor health service coverage and high mortality. Since then, Ethiopia has embarked on a massive effort to expand its health infrastructure and workforce into the rural areas where almost 80% of its population live. Two decades later, the gaps with other countries in sub-Saharan Africa in terms of maternal and newborn survival and other health indicators had reduced substantially. Estimated maternal mortality declined from 953 per 100 000 live births in 2000 to 267 in 2020, which was well below 536 for sub-Saharan Africa.^1^ Neonatal mortality declined from 48 to 27 per 1000 live births during the period 2000–2020, according to United Nation (UN) estimates, compared with a decline from 41 to 28 for sub-Saharan Africa.^2^

Ethiopia’s progress in improving health service has not gone unnoticed. Multiple studies have analysed Ethiopia’s progress and challenges.^3^,^9^ These include three ‘Exemplars in Global Health’ studies focused on learning from Ethiopia’s experiences (under-5 mortality, community health workers and stunting),^10^,^14^ the Countdown to 2030 country case study on multisectoral interventions and under-5 mortality,^15^ the global burden of disease,^16^ and a multiagency study on success factors for women’s and children’s health.^17^

There is broad agreement on a range of factors that contributed to Ethiopia’s health progress. Rapid national domestic economic growth, although from a low starting point, allowed for greater investments in the health and social sectors including the expansion of infrastructure. Government’s leadership to improve population health was strong, and global development partners were leveraged in support of the country’s strategic plans. A multisectoral approach with a health systems lens was central to addressing the social determinants of health, leading to improvements in nutrition, and water and sanitation over the past two decades.^15^

The current study aims to better understand the characteristics of progress in maternal and newborn mortality during 2000–2020 and identify priority areas for further reduction of maternal and neonatal mortality, as Ethiopia is still far from its national and international mortality targets. We used a three-pronged approach. First, we examined Ethiopia’s progress within a phase-specific transition model for maternal, stillbirth and neonatal mortality, to identify where Ethiopia has made progress, assess how generalisable its experiences are, and where future challenges lie. Second, we reviewed the multiple health system changes in Ethiopia and their relevance to maternal and newborn health (MNH). Third, we applied a geographic lens to assess the extent to which the changes in mortality, coverage and health system occurred in all administrative regions. The study was conducted as part of a study of seven countries with major progress in maternal and neonatal mortality (the MNH Exemplars study that includes Bangladesh, Ethiopia, India, Morocco, Nepal, Niger and Senegal).^18^

## Methods

### Data sources

The main data sources for quantitative analyses were five national Demographic and Health Surveys (DHS) conducted between 2000 and 2019 (all publicly available).^19^ We also used global databases on maternal, stillbirth and neonatal mortality,^2^ causes of neonatal death,^20 21^ the WHO’s Global Health Expenditure Database,^20^ socioeconomic indicators,^22 23^ Ministry of Health databases and reports with information on health infrastructure, workforce, policies and plans. National level estimates of maternal, stillbirth and neonatal mortality from the United Nations for 2000, 2010 and 2020 were used to compare Ethiopia’s change with other countries in sub-Saharan Africa.^1 2 24^ In addition, we synthesised data from health facility assessments in 2014, 2016 and 2018, and emergency obstetric and new-born care assessments in 2008 and 2016. An extensive review of literature and reports was used to gain further insights into the key characteristics of changes in MNH. Further details on the data sources and methods is available in online supplemental appendix.

### Analysis

For the mortality transition, we used a five-phase model for the maternal, stillbirth and neonatal mortality transition, which was developed for the MNH exemplars study.^25^ Phase I (maternal mortality ratio (MMR)≥700 per 100 000 live births, stillbirth and neonatal mortality rate combined (SBNMR)≥80 per 1000 births) indicates the highest levels of maternal and neonatal mortality, where access to services is extremely limited, inequalities are large, infectious diseases are a common cause of death, and fertility is high. In phase II (MMR 300–700, SBNMR 55–80), III (MMR 100–300, SBNMR 30–55) and beyond, infectious diseases and peripartum conditions (primarily obstetric complications and birth trauma) gradually become less prominent while conditions related to MNH status increase in relative importance, while coverage and quality of health services improve, inequalities in coverage change, and fertility declines. A country is considered to have reached the next transition phase if both mortality thresholds have been passed. We compared key indicators of mortality, fertility, intervention coverage, inequalities (for institutional birth coverage) and health systems for Ethiopia with typical characteristics in phases I, II and III to better understand key characteristics of progress, benchmark current progress and identify some of the prospective changes required to progress in the mortality transition. The typical values in the transition model are based on the observed median and IQR of countries in the specific phase during 2000–2020 which was based on 151 countries with population of at least 1 million in 2000. For Ethiopia, cause of death trend analysis was limited to neonatal mortality for which national trend estimates were available.^26^

We analysed trends in key MNH indicators at national and subnational levels using the DHS. National-level analyses focused on temporal trends and patterns in programme coverage, quality and mortality. For subnational analyses, we used the nine regions and two chartered cities, as these are used in national surveys, to examine trends and patterns.

In addition to conventional coverage indicators of antenatal care for pregnancy (at least one visit and four or more visits among live births in the 2 years preceding the survey) and delivery care (percent of live births in health facilities and per cent of live births with caesarean section in the 2 years preceding the survey), we also calculated a content-qualified antenatal care coverage indicator (referred to as ANCq) which is based on a score, which represents the 5 years preceding the survey, that considers frequency of contact with services and content of care including timing of first visit, number of visits and types of care received including blood pressure measurements, blood and urine samples collected, and receiving at least two shots of tetanus toxoid^27 28^ (online supplemental appendix C). In addition, we analysed trends in coverage of family planning (demand satisfied among currently married women with modern methods). Fertility decline can have a major impact on mortality by reducing the proportions of pregnancies and births in high-risk categories such as young maternal age and higher birth order. We applied a simple demographic decomposition method proposed by Jain to estimate the contribution of reduced fertility to MMR and neonatal mortality rate.^29^

We examined policy and system levers (distal drivers) that governments can use to improve programmes, including governance and policies, health financing and health workforce; and service levers including access and quality of care (intermediate drivers) to improve coverage and equity, and ultimately impact survival and well-being of mothers and newborns.^30^ The Countdown Policy and Programme timeline tool and the Health Systems and Policies Dashboard for Tracer Indicators tool were used to compile and analyse relevant policy, programme and health systems information.^31^

The regional analyses aimed to assess whether changes in mortality, coverage and health infrastructure and workforce had occurred in all regions, as evidence of full penetration of national policies. For mortality, we used survey data on neonatal mortality, as stillbirths had major and variable under-reporting, which is common in household surveys, and maternal deaths were too rare in the survey data for disaggregated analyses.^32^ Survey sampling weights and key sampling variables were used where available to account for complex sampling designs (details in online supplemental appendix). Family planning (demand satisfied by modern methods), at least one antenatal visit for pregnancy and institutional birth indicators were used to assess geographical disparities in access to and utilisation of MNH services over the two decades. Linear regression estimates from four surveys were used to estimate the 2019 family planning coverage of modern contraceptive use in the 2019 survey. The annual average rate of change (AARC) was used to describe progress made at different periods, with specific focus on changes prior to and after 2010. To assess coverage inequality between regions, we used equiplots to visualise and absolute difference between two extremes coverage to quantify the rate of change in narrowing gaps overtime. All survey analyses were carried out using Stata V.17.^33^

## Results

### Benchmarking the mortality transition

**Figure 1:**
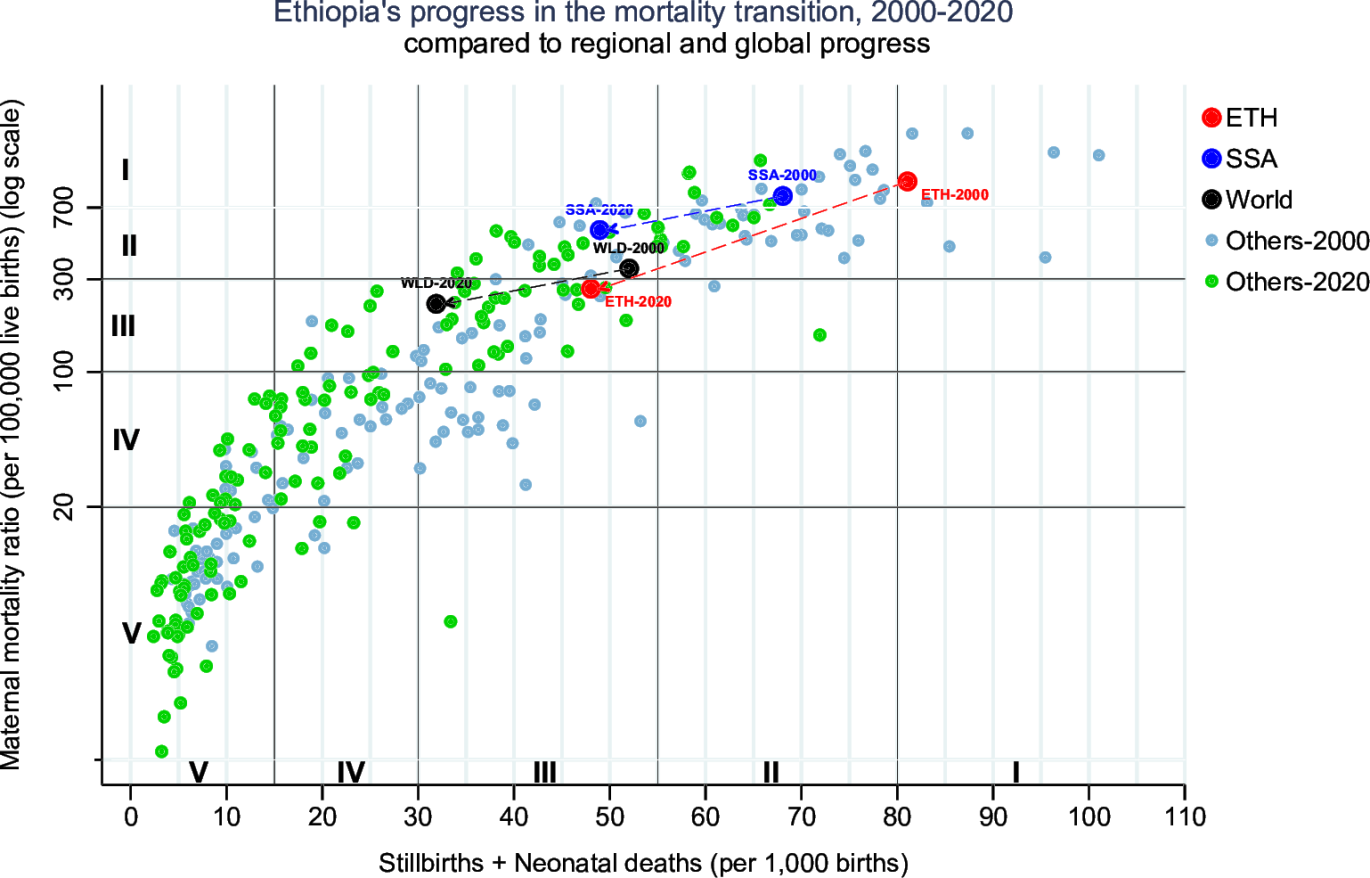
Maternal mortality ratio (per 100 000 live births) by stillbirth and neonatal mortality rate combined (per 1000 births), based on UN mortality estimates for 151 countries, with Ethiopia (ETH) (red dot), sub-Saharan Africa (SSA) (blue dot) and world (black dot) highlighted, 2000 and 2020. Others refers to the 149 countries with population of atleast 1 million. Each dot represents point estimates of mortality (ETH, SSA, World and 150 other countries) for 2000 and 2020.

All three mortality indicators showed major declines and a faster pace of decline during 2010–2020 than in the preceding decade, based on UN estimates. Maternal mortality declined from 953 to 635 and 267 per 100 000 live births in 2000, 2010 and 2020 (AARC: −4.1% and −8.7% for 2000–2010 and 2010–2020), respectively. Neonatal mortality decreased from 48 to 38 and 27 per 1000 live births in 2000, 2010 and 2020 (AARC: −2.4% and −3.4% for 2000–2010 and 2010–2020), respectively. Stillbirth rates decreased from 33 to 28 and 21 per 1000 births in 2000, 2010 and 2020 (AARC: −1.6% during 2000–2010 and −2.8% from 2010). Ethiopia progressed in the mortality transition from phase I in 2000 to early phase III in 2020 (figure 1). The Ethiopia mortality decline outpaced the overall decline in sub-Saharan Africa changing Ethiopia’s ranking from higher to lower than the regional average during 2000–2020 (figure 1 and online supplemental appendix figure 1). Ethiopia’s decline during 2000–2020 ranked fifth for maternal, and third for stillbirth and neonatal mortality combined among 42 countries in sub-Saharan Africa with a population of at least one million by 2000. For 2010–2020, Ethiopia ranked first among the same countries for both mortality indicators.

Table 1 summarises the key indicators for Ethiopia’s transition in 2000, 2010 and 2020, and compares these to typical values for core indicators in phases I–III of the mortality transition. The ratio of stillbirths and neonatal deaths to maternal deaths increased from 8 to 18 during 2000–2020, which is observed in most countries as maternal mortality has fallen faster than stillbirths and neonatal deaths. Ethiopia’s ratio was within the typical range for phase III.

**Table 1:** Key indicators of maternal and newborn health in 2000, 2010 and 2020 in Ethiopia, with the typical values (median and IQR) for countries in phases I, II and III of the mortality transition

Indicator	Ethiopia			Typical value		
	2000	2010	2020	Median (IQR)		
Transition phase	I	II	III	I	II	III
Mortality*						
Maternal mortality (per 100 000 live births)	953	634	267	≥700	300–700	100–300
Neonatal mortality (per 1000 live births)	48	38	27	≥45	30–45	15–30
Stillbirth rate (per 1000 births)	33	28	21	≥40	25–40	15–30
Ratio stillbirth+neonatal to maternal death	8	10	18	8 (6–9)	10 (9–12)	18 (15–25)
Cause pattern (neonatal) (% distribution)†						
Infectious conditions	34	27	25	28 (26–33)	20 (18–23)	16 (14–21)
Health and nutritional status (prematurity, etc)	39	37	46	44 (39–46)	52 (49–55)	58 (53–66)
Peripartum complications	27	26	29	28 (27–29)	28 (27–29)	26 (23–28)
Fertility‡						
Total fertility rate (total children per woman)	6.6	5.2	4.2	6.1 (5.8–6.9)	4.5 (3.9–5.0)	3.1 (2.3–4.3)
Adolescent fertility rate (per 1000 women)	112	83	67	102 (99–141)	96 (64–119)	67 (48–88)
Coverage of interventions§¶						
ANC4 or more visits (%)	9	17	44	44 (18–56)	51 (43–65)	69 (55–81)
Births in health facility (%)	5	12	54	36 (25–48)	59 (43–75)	74 (57–90)
Births in hospital (%)	2	4	13	19 (11–28)	26 (16–34)	48 (28–68)
C-sections (%)	1	2	7	2.3 (1.5–4.2)	4.5 (3.0–6.4)	12.9 (6.3–21.7)
Health inequalities§¶						
C-section, poorest quintile (%)	0.1	0.2	2.4	0.8 (0.3–1.7)	1.7 (0.9–2.7)	4.3 (2.1–10.7)
Delivery care, rural (%)	1.6	4.4	46	25 (19–40)	48 (33–68)	58 (44–81)
Delivery care rich—poor gap (abs. difference)	23	49	69	54 (36–61)	51 (38–62)	44 (21–63)
Health system characteristics**						
Total health expenditure per capita (US$)	5	16	29	42 (35–61)	44 (32–58)	74 (54–219)
Current health expenditure as % of GDP	4.7	4.5	3.5	5.4 (5.3–8.4)	4.1 (3.4–6.5)	4.7 (3.4–6.5)
Out-of-pocket expenditure on health (% of total expenditure)	36	42	33	59 (52–64)	42 (29–51)	31 (11–42)
Core health professionals (per 10 000 population)	2	5	12	52–10	85–12	20 (10–34)
Socioeconomic changes††						
Gross national income per capita (US$)	120	370	880	280 (200–400)	845 (540–1420)	1825 (1090–3405)
Secondary enrolment (gross), girls (%)	14	35	n/a	16 (12–24)	45 (37–53)	74 (47–80)

* UN IGME estimates.

† WHO/MCEE.

‡ UNDP estimates.

§ Data represent 2-year period preceding surveys (DHS conducted in 2000, 2011 and 2019 for Ethiopia). For detailed on the data sources see online supplemental appendix.

¶ DHS 2000, 2011, 2019.

** WHO Global Health Expenditure Database and Ministry of Health.

†† World Bank and United Nations Educational, Scientific and Cultural Organization (UNESCO).

ANC, antenatal care; DHS, Demographic and Health Survey; GDP, Gross Domestic Product; IQR, Interquartile range; n/a, not available; UN IGME, United Nations Inter-agency Group for Child Mortality Estimation.

The distribution of the three main groups of neonatal causes of death changed during 2000–2019 with decreasing prominence of infectious conditions, a similar share of peripartum conditions and increasing importance of causes related to the health and nutritional status of the newborn related to prematurity and congenital anomalies. Despite the decline in infectious disease mortality, Ethiopia still had a greater share of infectious disease mortality compared with the typical transition values in phases II–III.

**Figure 2:**
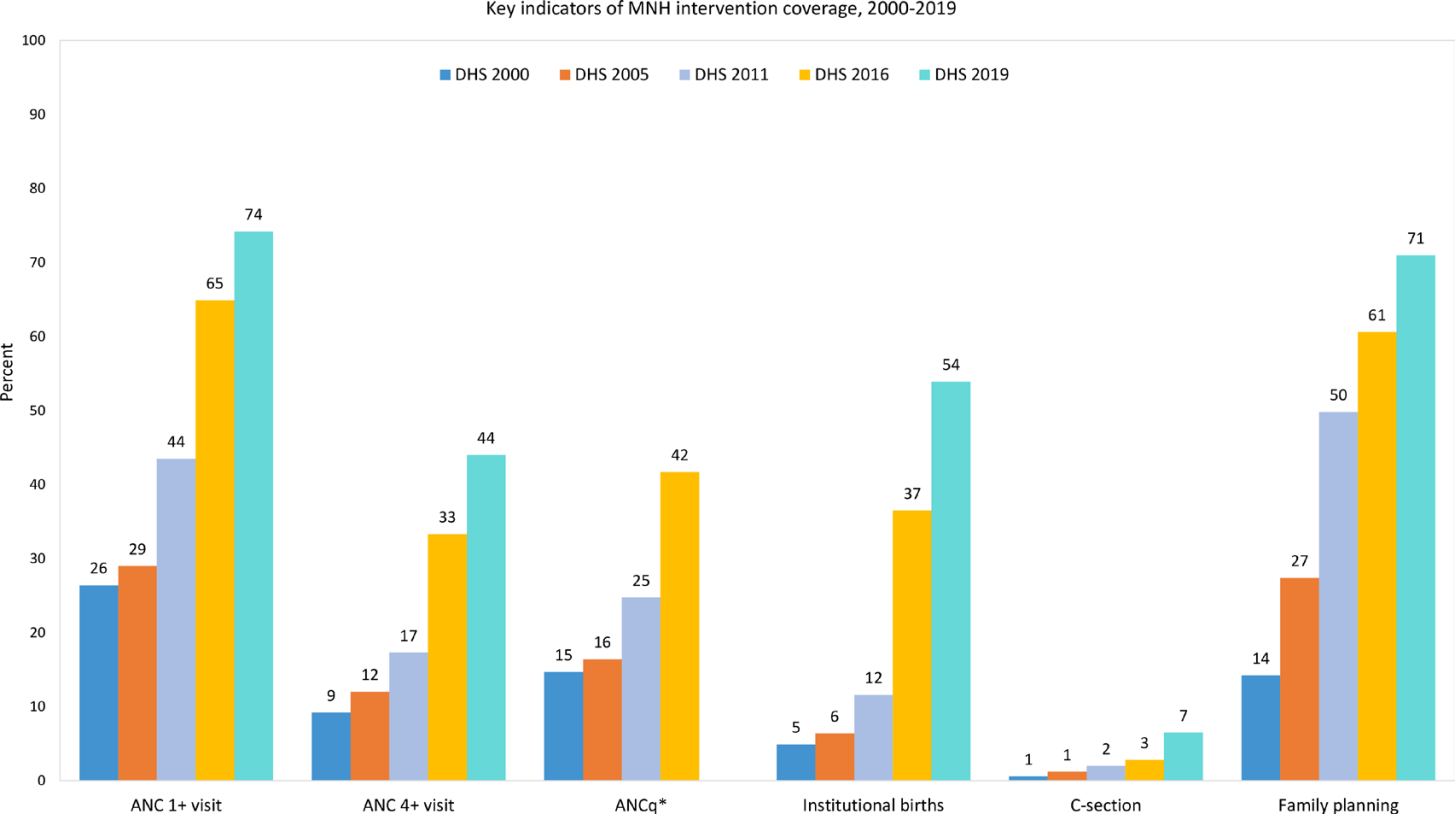
Trends in key indicators of antenatal care (ANC first visit and four or more visits, ANCq is with contents and timing)*, delivery care (institutional births and C-sections) and family planning (demand satisfied with modern methods among currently married women), Ethiopia DHS 2000–2019. DHS, Demographic and Health Survey; MHN, maternal and newborn health.

Total and adolescent fertility rates declined rapidly during 2000–2020. By 2020, Ethiopia’s fertility rates were within the typical range for countries in phases II and III. Total fertility declined from 6.6 to 4.2 children per woman during 2000–2020 (AARC −2.4% and −2.2% during 2000–2010 and 2010–2020, respectively). The fertility decline was associated with major increases in use of modern contraceptives by currently married women, resulting in an increase of demand satisfied with modern methods from 14% in 2000 to 27% in 2005, 50% in 2011 and 61% in 2016, according to the respective national surveys (figure 2). The increase in family planning coverage was fastest between the DHS surveys in 2000 and 2011 (AARC 11%) and slowed down after 2011 (AARC 4% during 2011–2019). Using Jain’s method, we estimated that 29% of both the MMR and neonatal mortality rate decline during 2000–2020, respectively, were associated with the decline in fertility.

Major increases in intervention coverage were observed for all MNH indicators. Basic contact coverage, as measured by the first ANC visit coverage, increased rapidly from about 2005, while coverage of four or more antenatal visits, institutional delivery coverage, and use of C-sections increased fastest from 2010 (table 1 and figure 2). For instance, AARC in coverage of births in health facilities increased from 8% to 19% between the surveys in 2000 and 2011, and 2011 and 2019, respectively.

The increase in institutional births was driven by lower-level health facilities which increased from 3% to 38% during 2000–2019. The institutional birth coverage (54% in DHS 2019), percentage of births in hospitals (13%) and the C-section rate (7%) were well below typical values in early phase III of the transition. The ANCq indicator, measuring antenatal care with contents coverage, increased from 15% in 2000 to 25% in 2011 and 42% in 2016, keeping pace with contact coverage indicators (figure 2).

The percentage of births delivered at health facilities in rural areas increased from 2% in DHS 2000 and 4% in DHS 2011 to 29% in DHS 2016 and 46% in DHS 2019 (table 1). C-section rates among the poorest increased from 0.1% in DHS 2000 to 2.4% in DHS 2019, which is indicative of large unmet need and lower than typical for a phase III country. The poorest to richest household wealth quintile absolute gap widened for institutional births between 2000 and 2019 which is observed in most countries during these phases of the transition. Further details on inequalities in MNH coverage indicators are presented in online supplemental appendix figure 4–8.

### Health system drivers of change

#### Governance

**Figure 3:**
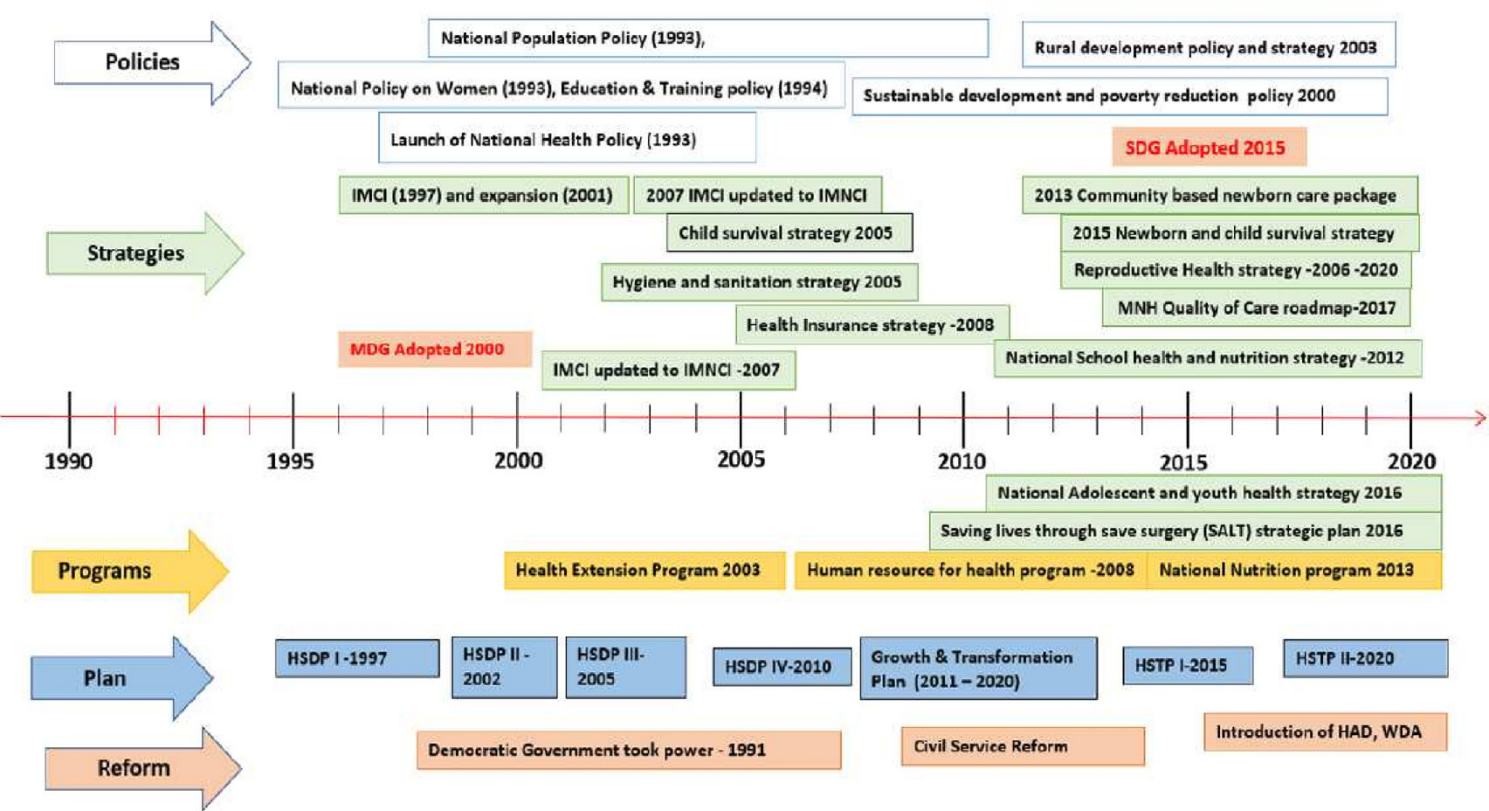
Timeline for key events, policies, strategies and programmes relevant to maternal and newborn health (MNH). IMNCI, integrtaed management of neonatal and childhood illness; IMCI, integrated management of childhood illness; SDG, Sustainable Development Goal; HSDP, Health Sector Development Plan; HSTP, Health Sector Transformation Plan; MNH, maternal and newborn health; HDA, Health Development Army; WDA, Women Development Army.

The policy timeline analysis indicated that maternal and child health was at the centre of the transformation of the Ethiopian health system during the last two decades (figure 3). Broad health and development aspirations were operationalised in specific national health strategies, including Health Extension Programme (HEP)–a programme launched in 2003 with 17 packages under four areas of focus (family health, disease prevention and control, hygiene and environmental sanitation, and health education communication)^3^–, policies and plans which were well aligned and based on international evidence and initiatives. Maternal health policies were prominent in line with the Millennium Development Goals. Newborn health issues became more explicit in plans from 2005 but were only prioritised from 2010 and expanded in subsequent years.

The alignment of development partners with the national framework has been a central element and was perhaps stronger than in other countries with budgets that rely on external funding. For instance, Ethiopia organised regular joint assessments of national strategies which aimed to align all development partners around a single plan, budget and monitoring system. Regular monitoring and progress reviews were a key feature of the system.

#### Health financing

Per capita total health expenditure increased from US$5 in 2000 to US$25 in 2020 but was well below typical values for phase III (table 1). Government health expenditure as a per cent of GDP peaked at 5.4% in 2010 but since decreased to 3.5% in 2020. Out of-pocket expenditure accounted for about one-third of total health expenditure, declining from a peak of 47% in 2011. External funding for MNH increased from US$0.47 per live birth in 2002 to US$1.23 in 2010 and US$3.11 in 2018.^34^

In 2013, delivery services in all public health facilities became free of charge, coupled with free ambulance services to facilitate referral when necessary.^35 36^ The community-based health insurance benefit package was launched in 2011, covering all outpatient and inpatient services at the health centre and hospital. In 2019, 28% of households were enrolled in the scheme.^37^

#### Health workforce

**Figure 4:**
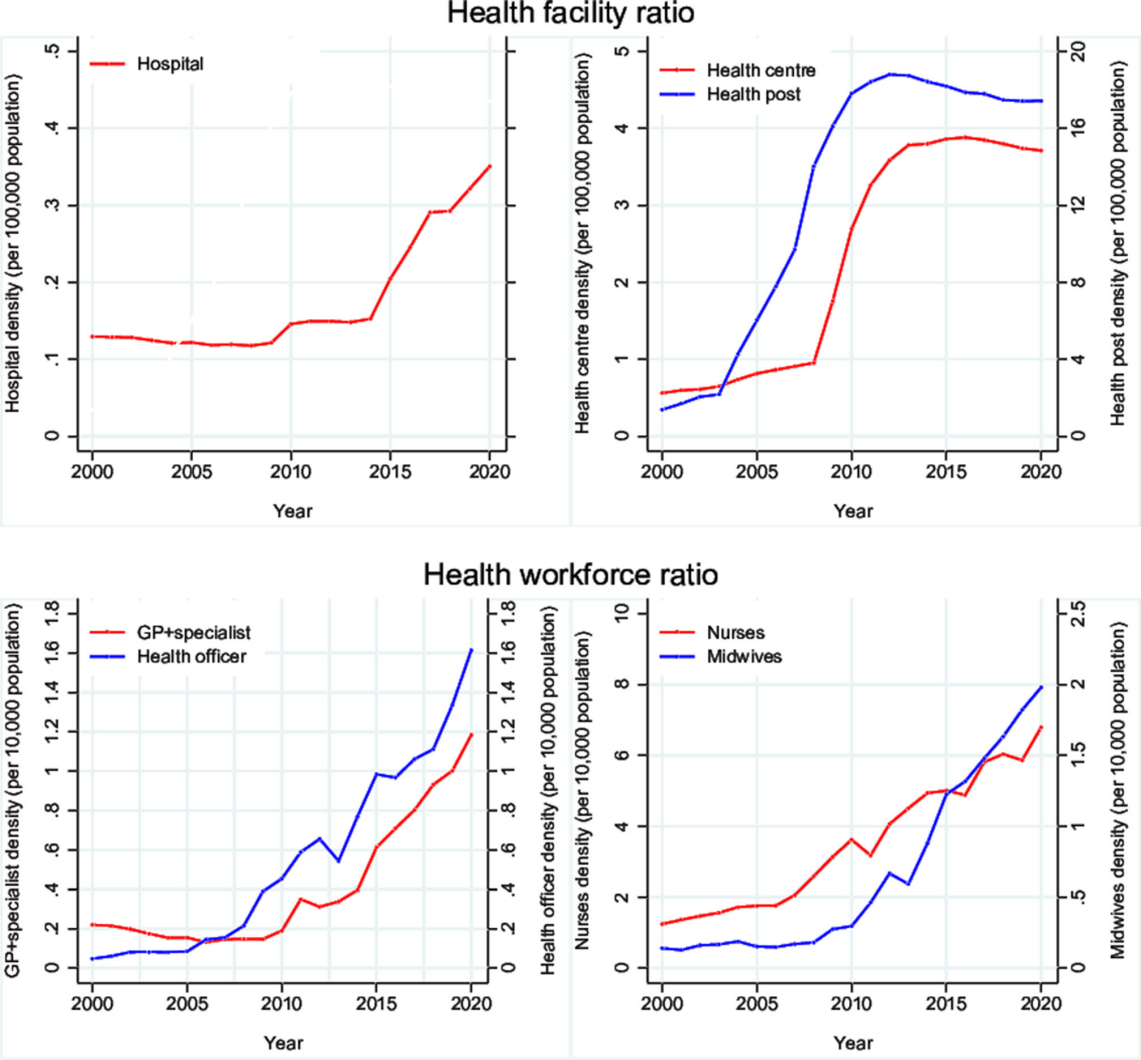
Trends in health workforce (by 10 000 population) and health facilities (by 100 000 populations), Ethiopia, 2000–2020. GP, general practitioner.

Starting from a very limited base, especially in rural areas, Ethiopia used a multipronged strategy to expand the health workforce, create demand for services and bring health service providers closer to the people. Training and deployment of health professionals was stepped up from 2003 which led to large increases in the density of nurses and midwives from 2008 to 2010 and physicians from 2014 (figure 4). The number of midwives per 10 000 population increased from 0.1 in 2000 to 0.3 in 2010 and 1.2 in 2020. Overall, the density of core health professionals (physicians, nurses, midwives and health officers) increased eightfold to 12 per 10 000 population during 2000–2020, especially from 2010, reaching typical values for phases II and III of the mortality transition (table 1). The absolute increase during 2010–2020 was 7 per 10 000 population, more than double the increase during 2000–2010 (3 per 10 000 population).

The creation of the Integrated Emergency Surgery and Obstetrics (IESO) cadre of non-physician surgical providers in 2009 was a critical adaptation to improve access to C-section in rural areas as part of a task shifting approach. By 2015, the new cadre of IESO were performing emergency obstetric and other surgical procedures at 800 primary level hospitals.

There was also a rapid scale-up between 2005 and 2009 of Health Extension Workers (HEWs). HEWs are salaried government employees—assigned to health posts on completion of a 12-moth training—working directly with households and their focus include mapping households to prioritise health issues and draft action plans.^3 10^ Health posts are staffed by two HEW who provide preventive, promotive and basic curative services in addition to community-based activities for populations of 3000–5000 people, including promotion and provision of basic antenatal, delivery and postnatal by 2009–2010, there were about four HEW per 10 000 population, with densities stabilising after that.

In 2011, when service coverage was still below targets, further efforts were made to enhance community demand for services through volunteers supervised by the HEWs, known as the Women’s or Health Development Army (HDA)—a women-centred community organisation that requires the establishment of teams of up to 30 households with 5 subgroups of 6 members (referred to as one-to-five networks)—covering close to 13 million households by 2013.^3^ Under the supervision of HEWs, HDAs’ responsibilities include identifying local bottlenecks that hinder families from utilising key services and implementing the HEP, and help HEWs in prioritising local issues that they want to address as a team, as well as coming up with feasible strategies to address identified problems and evaluate their activities.^3 38^ Most importantly, promoting use of maternal, newborn and child health services are central part of their activities, and reports have shown that the volunteer army coverage reached at almost universal level in agrarian settings and partial coverage in urban settings.^39^ Several studies have concluded that the volunteer army contributed significantly to the increased uptake of services.^4 40^ Despite their contribution in improving MNH, the 2019 national HEP assessment highlighted that challenges still exist to fully use HDAs’ structure for community engagement (such as due to low capacity and acceptability) and suggested for alternative engagement options for HEP (such as introduction of innovative motivation mechanisms for community volunteers).^39 41^

#### Access to services

Access to antenatal and delivery care increased from 2003 onwards, as part of the HEP. The expansion of health infrastructure started with health posts in 2003, reaching the rural communities with almost one health post per 5000 population by 2012 (figure 4). The density of health centres increased rapidly during 2008–2012 to about one health centre per 27 000 population. The expansion of hospitals only took off around 2014, tripling the numbers over the period 2010–2020. The increase in static facilities was accompanied by the provision of one or two ambulances to all districts, reaching 4011 of which 85% were functional in 2021, and by a fourfold expansion of the paved road network over two decades.

Access to services was further promoted through the establishment of maternity waiting homes within or around health centres and hospitals. Ministry of Health guidelines for national expansion of maternity waiting homes were issued in 2015. By 2016, 56% of health centres and 27% of primary hospitals had maternity waiting homes.^42^ No national data were available before the 2016 assessment. Several studies in Ethiopia have documented a positive impact of maternity waiting homes on skilled birth attendance and health outcomes.^34^

Reports from facility assessment surveys also indicated that nearly all health centres and hospitals offered delivery services during 2008–2018. Availability of neonatal bag and mask—a tool for neonatal resuscitation^43 44^—increased from 40% in 2008 to 73% in 2016. Yet, coverage of specific interventions such as kangaroo mother care and neonatal resuscitation was still low according to facility assessments in 2016 and later.^14^ Nearly half (49%) of neonatal intensive care unit (NICU) in hospitals, an initiative first started in 2010, became functional by 2015, and the number of hospitals with NICUs increased from 30 in 2015 to 196 in 2021. Further details on selected tracer services and items from facility assessment are presented in online supplemental appendix tables 5 and 6.

Health facility assessment in 2008 and 2016 showed that the increase in the numbers of hospitals and health centres was accompanied by modest increases in the availability of emergency obstetric and newborn care (EmONC) in those facilities. The percentage of hospitals with comprehensive EmONC increased from 51% to 62%. Among health centres, 7% were functioning as basic EmONC centres in 2016, up from just 1% in 2008.^42^ Service readiness improved for tracer items such as magnesium sulphate, oxytocin and bag and mask for neonatal resuscitation, and hospital capacity for C-section and blood transfusion, though major gaps remain especially for neonatal care of small and sick newborns (online supplemental appendix table 6).

### Subnational analysis

**Figure 5:**
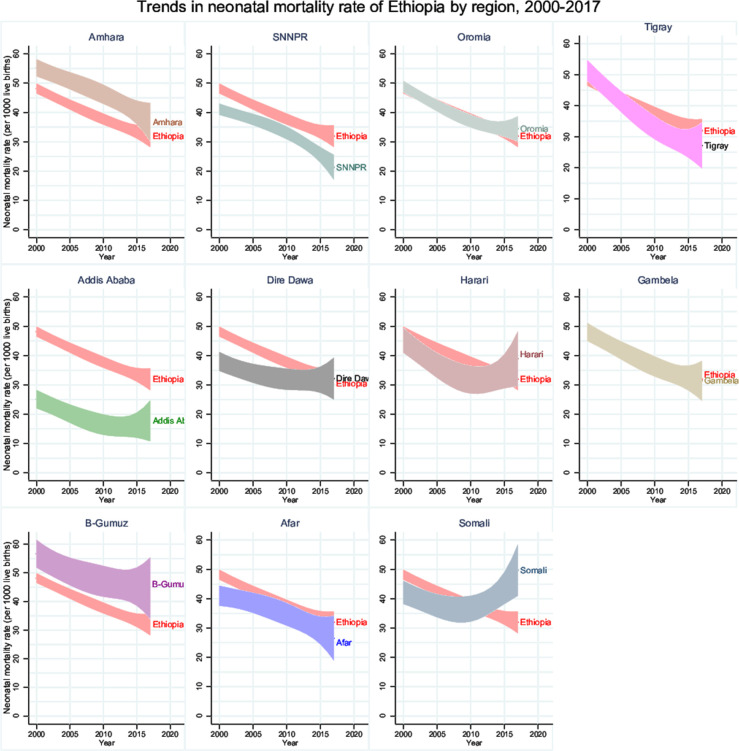
Trends in neonatal mortality rate (per 1000 live births) of Ethiopia by region, 2000–2017. SNNPR, Southern Nations, Nationalities and People’s Region; B-Gumuz, Benishangul-Gumuz.

Figure 5 presents neonatal mortality estimates by region, based on all DHS survey data. All regions except Somali had considerably lower neonatal mortality in 2017 than in 2000, with the largest declines (by nearly 50%) observed in Tigray and Southern Nations, SNNPR regions and smallest in Harari, Somali and Dire Dawa regions during 2000–2017. However, the declining pattern of mortality before and after 2010 was mixed; neonatal mortality rate in Harari and Somali after 2010 may exhibit an increasing trend while there exists little or no change in Addis Ababa, Benishangul Gumuz and Dire Dawa regions during 2010–2017. Mortality in Oromia and Amhara regions—the two most populous regions—decreased slower than the national rate. The large CIs around the neonatal mortality estimates preclude an accurate comparison of rates of decline before and after 2010 by region.

**Figure 6:**
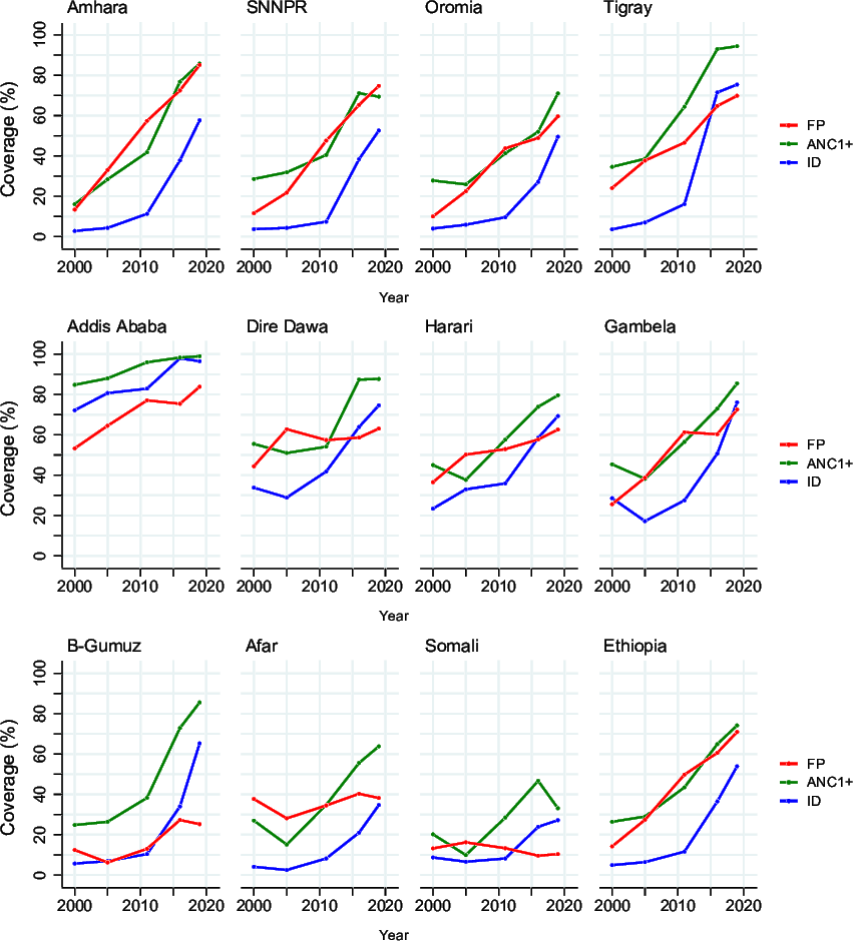
Key indicators of maternal and newborn health intervention coverage by region, DHS surveys in 2000, 2005, 2011, 2016 and 2019. ANC1+, antenatal visits for pregnancy: 1+ visits; ID, representing place of delivery at health facility; FP, representing demand for family planning satisfied by modern methods (the 2019 data point for FP was linearty inputed using four previous rounds of data). ANC, antenatal care; DHS, Demographic and Health Survey; FP, family planning; ID, institutional delivery; SNNPR, Southern Nations, Nationalities and People’s Region; B-Gumuz, Benishangul-Gumuz.

The trends in intervention coverage in the three largest population regions—Amhara, Oromia and SNNPR—and Tigray region exhibited almost a similar pattern during 2000–2020, presenting earlier and faster family planning coverage increases (prior to DHS 2011) and later acceleration of coverage in maternal and newborn interventions (notably during post-DHS 2011). The national pattern of earlier increases in family planning coverage and later increases in MNH interventions coverage was evident in most regions (figure 6). The two pastoralist regions Somali and Afar stood out with much lower than national coverage and slower progress on all indicators, especially family planning. Geographical disparities in selected MNH coverage persist during 2000–2019 (online supplemental appendix figure 9 and online supplemental appendix table 3). The pace of decline of fertility rate was faster in Amhara region (reduced at an average rate of 3.3% per year) than any other region. The two extremes were Addis Ababa with a very low total fertility rate (2.2 in 2000 and 1.9 in 2018) and the pastoralists regions, with fertility estimated at 6.4 in Afar and 7.5 in Somali in 2018—almost similar to the rate in 2000 (6.7 in Afar and 8.3 in Somali). Details of the changes over time for individual coverage indicators and fertility are shown in online supplemental appendix tables 2 and 3.

Health workforce densities increased dramatically in all regions, and more so post-2010 than in the preceding decade in most regions (online supplemental appendix table 4). Amhara and SNNPR had absolute increases similar to the national increase of 10 core health professionals per 10 000 population, while Oromia’s increase was considerably smaller (6 per 10 000). The greatest increase in the overall density of core health professionals occurred in Tigray and Addis Ababa, and several other regions with small populations. By 2020, health workforce densities in Amhara and SNNPR, as well as in Somali, were close to the national levels, while Oromia and Afar were well below.

## Discussion

Ethiopia has made remarkable progress in MNH during 2000–2020. Maternal and neonatal mortality dropped by 72% and 44%, respectively, fertility declined by more than one-third, antenatal care coverage increased almost three times and delivery care coverage increased more than ten times, accompanied by major improvements of its health system indicators. Ethiopia was among the top five performers in sub-Saharan Africa, and it experienced the fastest decline during 2010–2020 for both maternal mortality and for combined stillbirth and neonatal mortality. Ethiopia was one of six countries (among 116 that were in phases I–IV in 2000) in the world that progressed more than one phase in the mortality transition.^25^

Starting from a weak health infrastructure, low coverage and limited resources, Ethiopia prioritised expanding the basic infrastructure and health workforce to the vast rural population, while generating demand for services. The fastest mortality declines and main intervention coverage increases occurred after 2010. Even though the HEP was launched in 2003, resulting in major expansion of the basic health infrastructure, initially through health posts, and rapid training and deployment of HEWs, the impact on coverage was modest by 2011, especially in rural Ethiopia. This can be attributed to several factors. First, investments of the health infrastructure and workforce for MNH services take several years before they result in increased access. From about 2008, the investments in training started to pay off and the density of core health professionals including midwives started to increase rapidly, health centre availability doubled, and availability of emergency obstetric and neonatal care improved. Second, demand generation received limited attention during the initial rapid expansion of HEWs.^45^ After 2010, community mobilisation and demand generation through the HDA became an important contributing factor for increased service uptake. Third, several initiatives contributed to further increase in access such as the ambulance programme, the maternity waiting homes expansion and the deployment of a new cadre of surgeons which became operational at scale from about 2015, resulting in an increase in C-section rates from about 2% in 2016 to 5.4% in 2019. Furthermore, neonatal health became a government priority from 2010 onwards. Given these health system developments, it is not surprising that the neonatal mortality average annual rate of decline from 2010 onwards was almost two times greater than in the preceding decade. Fertility decline may have been an important factor of the maternal and neonatal mortality declines prior to 2010 and during 2010–2020.

The subnational analysis provided further insights into the progress in Ethiopia. Broadly, there have been major improvements in all regions, often from very different baseline levels in 2000. All regions had major increases in coverage of MNH interventions, starting in 2005 and greatly accelerating from 2010. In most regions, this was preceded by increases in family planning coverage. National change was driven by the three large population regions of Oromia, Amhara and SNNPR, accounting for about 80% of neonatal mortality. There are, however, large differences between the regions in mortality patterns and service coverage. Somali and, to a lesser extent Afar, are still well behind all other regions on most indicators. Regional inequalities have not reduced.

Based on results from our phase-specific transition model for maternal, stillbirth and neonatal mortality, Ethiopia has advanced from the highest mortality phase I to early phase III. The Sustainable Development Goal (SDG) targets for 2030 are located in the middle of phase IV. For several indicators, such as fertility and health workforce density as well as intervention coverage inequalities, Ethiopia’s progress was typical for a country moving from the highest mortality phase I to early phase III. Coverage of antenatal and delivery care was however still lower than typical phase II values and major increases are needed to move through phase III. Inequalities are large between household wealth, urban–rural residence and administrative regions which is typical in the early phases of the transition but should start to decrease rapidly in phase III. In addition, the main increase in delivery coverage was driven by health centres and health posts, and not by hospitals where the chances of receiving comprehensive emergency obstetric and neonatal care are much greater. The same applies to total health expenditure per capita and the government contribution of current health expenditure which were both lower than typical values in phase II and far from phase III.

An organised system of health strategies, policies and plans has been in place since the late 90s, with reviews of progress, financing reforms and accountability. Key characteristics were the generation and use of evidence for programmes, prior to scaling up (eg, extensive research on the HEP,^3 4 9^ and community newborn and child health^46^), as well as a system of joint reviews with development partners to monitor progress and improve planning. A comprehensive health systems lens, rather than a collection of vertical programmes, was applied to the HEP which became the main vehicle to increase coverage of the essential services for MNH and several other primary healthcare services. Others have argued that the multisectoral approach was an important driver of progress in especially child survival.^15^ The well-coordinated government-led mobilisation and use of external funding have been identified as one of the drivers of success in Ethiopia, despite the high dependence on external aid.^47 48^ These are all aspects of a learning health systems approach.^49 50^

The key indicator values that characterise the next phase of the mortality transition reveal major challenges and opportunities for Ethiopia. For many indicators, the pace of progress needs to be maintained, including coverage increases of life-saving interventions, health system strengthening and improved subnational data for decision-making. Increasing coverage of essential interventions among disadvantaged populations, such as rural women and newborns, and the poorest households, is critical. Major reductions in inequalities must occur when transiting through phase III and into IV, implying continuous monitoring of progress and performance of the health system with an equity focus is warranted.

One of the limitations of our study, as presented here, is the omission of a detailed analysis of non-health sector changes that could affect maternal and newborn mortality. As multiple studies have highlighted, including our own Exemplar study report,^30^ socioeconomic changes resulted in more households coming out of poverty, access to electricity, water and sanitary facilities and means of transport and communication. This undoubtedly has contributed to major progress in the rural areas, even though many economic indicators were still at a low level by 2020, as also shown in the comparison with other countries in phases II and III of the mortality transition. In addition, important social changes occurred as women became more literate and educated, gradually getting married later, had fewer children and experiencing some increases in agency and autonomy. These economic and social changes are likely to contribute to improved survival of mothers and newborns in Ethiopia but quantifying their contribution versus the health sector is complex, given the multitude of pathways in which maternal and newborn survival can be affected.^30^ In addition, we did not attempt to quantify the contribution to mortality declines of specific interventions or programmes since most policy and programme changes occurred concurrently, pathways are complex, and mortality estimates have substantial uncertainty at the regional level (and even the national estimates).

Despite the significant progress, Ethiopia still has a long way to go through the maternal, stillbirth and neonatal mortality transition and reach SDG targets. Universal coverage of MNH care can only be achieved by reaching all women and newborns in all regions and by ensuring the poorest and rural populations have access to services. Evidence-driven focus on the largest population regions where major gains can still be made, especially Oromia, SNNPR and Amhara, is a necessity for continued major national progress. Further expansion of health infrastructure and health workforce, combined with further innovative and data-driven approaches, especially in the nomadic regions, are necessary. Dealing with the adverse consequences of the recent civil war on maternal and newborn survival is also critical. Finally, increasing the quality of care and ensuring universal access to emergency obstetric and neonatal obstetric care will increasingly require greater investments in hospitals, midwives and physicians, referral networks, maternity waiting homes and other investments to ensure that every woman and baby have access to life-saving interventions.

## Supplementary material

10.1136/bmjgh-2023-011911 online supplemental file 1

## Data Availability

All data relevant to the study are included in the article or uploaded as online supplemental information.
