# Supplementary material for: Learning from Ethiopia’s success in reducing maternal and neonatal mortality through a health systems lens
Source: BMJ Glob Health. 2024 May 6;9(Suppl 2):e011911. doi: 10.1136/bmjgh-2023-011911 (PMC11085893; doi:10.1136/bmjgh-2023-011911)
Supplement: online supplemental file 1 [file bmjgh-2023-011911supp001.pdf]

## Appendix: Data sources, analytical methods and more results

### A. Data sources

This section first describes the data sources used for the assessment of the mortality transition in Ethiopia in comparison to typical values for phases I, II and III as shown in Table 1 of the main paper. The second part summarizes the data sources used for further analyses on health systems and regions.

The data sets used to ascertain Ethiopia's indicators against the medians and interquartile ranges for the maternal, stillbirth and neonatal mortality transition model were the following:

***Mortality:*** Maternal mortality ratios (MMR) were extracted from the WHO database for 2000 to 2020. https://www.who.int/publications/i/item/9789240068759 (accessed 23 February 2023). Details of the methods used by the UN are provided in World Health Organization.[1] It is defined as:

- *Maternal mortality ratio:* the number of maternal deaths in a population per 100,000 live births during one calendar year.

Stillbirth rates and neonatal mortality rates were extracted from the UN Interagency Group for Child Mortality estimation (IGME) database (https://childmortality.org/) (accessed 12 December 2022). The following definitions are used (https://childmortality.org/methods).[2]

- *Stillbirth rate:* the number of babies born with no sign at life at 28 weeks or more of gestation per 1,000 births, each year
- *Neonatal mortality rate:* the number of deaths of infants under 28 days of age per 1,000 live births, each year

We computed defined stillbirth + neonatal mortality per 1,000 births as: stillbirth rate per 1,000 births + neonatal mortality rate per 1,000 live births/(1+stillbirth rate/1000)

***Socioeconomic indicators:*** Gross National Income (Atlas method, current US$) was extracted from: https://data.worldbank.org/indicator/NY.GNP.PCAP.CD (accessed 24 February 2023)

School enrollment, secondary (% gross) from : https://data.worldbank.org/indicator/SE.SEC.ENRR (accessed 24 February 2023) Gross enrollment ratio is the ratio of total enrollment, regardless of age, to the population of the age group that officially corresponds to the level of education shown. Secondary education completes the provision of basic education that began at the primary level and aims at laying the foundations for lifelong learning and human development, by offering more subject- or skill-oriented instruction using more specialized teachers.

***Causes of death – neonatal:*** Details of the cause of death analysis are presented in Annex D. The causes of neonatal death were obtained from the WHO Global Health Observatory. https://www.who.int/data/gho/data/indicators/indicator-details/GHO/distribution-of-causes-of-death-among-children-aged-5-years-(-) (accessed September 1 2022) These estimates were used to explore on causes of maternal and new-born deaths at national level, and they were triangulated further with data extracted from review of reports and literature.

---

[1] World Health Organization. Trends in maternal mortality 2000 to 2020: estimates by WHO, UNICEF, UNFPA, World Bank Group and UNDESA/Population Division. Geneva: WHO; 2023.

[2] United Nations Interagency Group for Child Mortality. Levels and trends in child mortality: report 2022. Estimates developed by the UN IGME. 2023.

***Fertility:*** National level estimates of total fertility rates and age-specific fertility rates (15-19 years) were extracted from: United Nations, Department of Economic and Social Affairs, Population Division (2022). World Population Prospects 2022, Online Edition. https://population.un.org/wpp/Download/Standard/MostUsed/

For total fertility rate (15-49) at national and subnational level, all five rounds of DHS survey datasets were used to retrospectively compute empirical values of total fertility rates by 3-year interval over 15-years period preceding each survey. This generates 5 empirical data points from each survey, leading to data since 1990s. Geographic and socioeconomic characteristics mainly area of residence, wealth quintile and geographic region were used for subnational analysis to understand inequalities in fertility over the past three decades. Missing data was used.

***Health financing:*** One are we explored was financial resources for MNH, mainly to assess health resource allocation. In addition to Ethiopia's National Health Account, WHO's Global Health Expenditure Database – a database which provides internationally comparable data on health spending for close to 190 countries from 2000-2017 was used to assess the levels and trends in domestic and donor finding flows for MNH. Data extracted from the WHO Global Health Expenditure database were from: https://apps.who.int/nha/database/Select/Indicators/en (accessed 25 Feb 2023). No data were available for DPRK, Somalia and the State of Palestine, 148 countries remained for our analyses. No data for 2020 were available from Albania, Saudi Arabia, Libya, Yemen and Syria. Afghanistan, Iraq, South Sudan and Zimbabwe had no data for 2000.

***Health workforce and facilities:*** Global Health Workforce Statistics, The National health Workforce Accounts database, World Health Organization, Geneva. https://www.who.int/data/gho/data/themes/topics/health-workforce (accessed 28 Feb 2023). Because there are a substantive number of missing annual values, we used period rates as there were many years with missing data and present those as 2002 (2000-2004) and 2018 (2016-2020), matched to the transition phase in those years. As for Ethiopia, annual review reports were retrieved and used to collate and synthesize data on health workforce and facilities.

***Coverage and inequalities: household surveys:*** For service coverage indicators, including inequalities, and neonatal mortality rates by place of birth, we analyzed data from all national Demographic and Health Surveys (DHS) and Multiple Indicator Cluster Surveys (MICS) conducted during 2000-2020. The analyses were done by the International Center for Equity in Health at the Federal University of Pelotas, Brazil. https://equidade.org/ Of the 326 household surveys, 38 were conducted when the country was in transition phase I, 126 in phase II, 71 in phase III, 76 in phase IV and 15 in phase V. The surveys were conducted in 99 low- and middle-income countries.

For further analyses of specific indicators beyond the transition model and for analysis of regional levels, trends and differentials we used the following data sets for **Ethiopia**:

- ***Household surveys:*** Ethiopia Demographic and Health Surveys (DHS) in 2000, 2005, 2011, 2016 and 2019, with each survey having data on a five-year recall period.
- ***Facility assessments:*** Emergency obstetric and newborn care assessments (EMONC) in 2008 and 2016, and Service Availability and Readiness Assessment (SARA) in 2016 and 2018, as well as Service Provision Assessment (SPA) in 2014.

## B. Description of models used to estimate neonatal mortality and total fertility rate

Below are the steps taken to generate point estimates that were used to fit with the model to determine the level and trends of neonatal mortality and total fertility rate at subnational level.

### *B.1. Point estimate analysis: neonatal mortality rate and total fertility rate:*

1) *Neonatal mortality analyses:* Empirical estimates of neonatal mortality were computed at national and subnational level, and by age group, socio-economic status and rural/urban residence, where appropriate. All five rounds of DHS and mini-DHS survey datasets were used to retrospectively compute the levels and trends of maternal and neonatal mortality NMR (in terms of stillbirths, and neonatal mortality in first week after birth, and in first 28 days) since 1990s. In addition to empirical mortality estimates, national level estimates of MMR and NMR available elsewhere (such as by UN-MMEG for MMR and UN-IGME for NMR) were also used to explore overall trends and changing patterns of mortality over the study period.
2) *Total fertility analyses:* All five rounds of DHS survey datasets were used to retrospectively compute empirical values of total fertility rates (national and subnational) by 3-year interval over 15-years period preceding each survey. This generates 5 empirical data points from each survey, leading to data since 1990s. Geographic and socioeconomic characteristics mainly area of residence, wealth quintile and geographic region were used for subnational analysis to understand inequalities in fertility over the past three decades.

For all empirical analyses using surveys, survey sampling weights and key sampling variables were used where available to account for complex sampling designs. The sampling weights are applied to point estimates while stratification is taken into account when calculating stands errors.

### *B.2. Model estimation of levels and trends of neonatal mortality and total fertility:*

A priori knowledge including pre-2000 data points were used to assign values for missing NMR and TFR data points for the period 2000-2017. For missing data points between 2000 and 2017 (excluding the two extremes), multiple imputation technique was utilized to estimate neonatal mortality and total fertility data points in a sequence of values. This method, described below, was used for all subnational estimation with at least one missing data points between 2000 and 2017. The method was used to estimate 9 pre-2000 missing data points and 10 post-2000 data points. We located all empirical estimates from survey in the middle of the reference period, taking into account the period of data collection and the length of the reference period. For instance, for a survey conducted in 2019 and the reference period for the mortality and fertility was five and three years, we used 2016.5 and 2017.5 as the year for the mortality and fertility data points, respectively. Similar approaches have also been used elsewhere[3].

A penalised B-Spline regression model that used the Markov Chain Monte Carlo (MCMC) method of multiple imputations for missing data was used to estimate both national and subnational levels and trends of neonatal mortality rate per 1000 live births and total fertility rate – the number of births per woman, and uncertainty from 2000 to 2018. In order to better account of random and systematic biases of survey data, we utilized multiple imputation technique using multivariate normal distribution assumption to impute missing data so that multiple data values generated from three different methods were imputed rather than a single value from a regression line to reflect the uncertainty range around the true value. The number of imputations used for each method were 100, with missing data points assumed as missing at

[3] Boerma T, et al. Global epidemiology of use of and disparities in caesarean sections. *Lancet*, 2018. 13;392(10155):1341-1348.

random. Variables describing regions and time were considered as covariates as appropriate for national level estimate and triangulate with other data sources (such as UN-IGME).

Trends of neonatal mortality and total fertility rate at subnational level were estimated with the penalized B-splines of degree 3 with 3 knots in order to model the regression function. Knot locations were decided by the models. For each mortality and fertility estimates, the spline regression models were fitted to all data inputs in each region, area of residence and wealth status (i.e., region-year data points). Note that the penalized B-spline function is typically much smoother than a model using spline transformation or a B-spline expansion since the changes in the coefficients of the basis are penalized to make a smoother fit[4]. For each analysis, the dependent variable is specified with an identity transformation and analyzed as is (i.e., with no transformations). The independent variable, year, is specified with penalized B-spline transformation, so a penalized B-spline model was fit. Furthermore, Schwarz Bayesian Criterion (SBC) along with smoothing parameter, requesting for a penalized B-spline analysis minimizing the SBC criterion, was utilized to produce smoother estimates. Uncertainty ranges around the estimated value were obtained from the penalized B-spline regression model.

Our estimation did not extend beyond 2017 for neonatal mortality and 2018 for total fertility as the most recent survey used to compute data points was DHS 2019, and neonatal mortality and fertility was computed by a five- and three-year period preceding each survey, respectively. Similar approaches have been utilized to estimate the levels and trends of neonatal mortality and fertility over the past three decades utilizing all available surveys collected since 2000.

As a triangulation exercise, estimates generated using this model was compared with data from UN-IGME estimates of neonatal mortality and UN estimates for total fertility rate (Appendix Figure 2). Though UN-IGME estimates use more empirical data points that this study, results shows that the two estimates are very much close such that estimates from this B-spline model and the 95% uncertainty interval falls within the 90% uncertainty interval of UN-IGME estimate. Similarly, TFR estimates from the B-spline model is much closer to the UN estimate. It is important to note the number of underlying data points and a prior information used in these models are different. As a limitation, our estimation approach did not adjust for crisis and HIS/AIDS, as well as no data from vital registration was used to adjust estimates. The analysis was conducted using STATA version 17[5].

***B.3. Fertility impact analyses:***

Jain's decomposition approach was utilized to decompose the overall decline in the overall MMR observed between 2000-2020 and NMR observed between 2000-2020 and quantity the decline attributable to fertility and safe motherhood program[6]. The decline in fertility, assessed both in terms of decline in crude birth rates and changed in age-parity birth risk composition – a proxy measure to estimate the effect of safe motherhood programs. Crude birth estimates from the United Nations, MMR from UN-MMEIG and NMR from UN-IGMEG were utilized to estimate the impact of fertility.

## C. Content qualified ANC indicator (ANCq)

ANCq is a survey-based ANC indicator calculated as a score, composed of seven variables which add points to the score: first visit in the first trimester of pregnancy (1 point), at least one visit with a skilled provider (2 points), total number of visits (1 point for 1–3 visits, 2 points for 4–7 visits, and 3 points for 8 or more visits), blood pressure measured (1 point), blood sample collected (1 point), urine sample

[4] Eilers, PHC. and Marx, B.D. Flexible Smoothing with B-splines and penalties. Statistical Sciences; 1996; 11(2): 89-121.
[5] StataCorp. 2021. *Stata Statistical Software: Release 17*. College Station, TX: StataCorp LLC.
[6] Jain AK. Measuring the effect of fertility decline on maternal mortality ration. Studies in Family Planning. 2011;42(4):247-60.

collected (1 point), and receiving at least two shots of tetanus toxoid (1 point). Thus, the ANCq score varies from zero, for women with no ANC, to 10 points, for women getting top points for each item. ANCq was validated using a convergent validation exercise exploring the association with neonatal mortality, where higher scores of ANCq were associated with lower neonatal mortality. Full details on the construction of the indicator and its validity are presented elsewhere.[7]

## D. Health facility assessment

These surveys were utilized to assess service availability and readiness/preparedness to provide quality MNH-specific services. This helps to understand the extent to which the policies and plans led to increased service availability, utilization and quality can partly be assessed through the health facility assessments. Therefore, data from three rounds of facility surveys (one round of Service Provision Assessment (SPA), conducted in 2014, and two other surveys of Service Availability and Readiness Assessment (SARA), conducted in 2016 and 2018), as well as two rounds of EmONC survey conducted in 2008 and 2016 were utilized for assessing service availability and readiness/preparedness to provide quality MNH-specific services. Data were collated and used to assess progress on selected tracer services and items relevant for MNH. Furthermore, annual review reports were retrieved and used to collate data on health system inputs and output indicators, while other review of documents such as on maternal death surveillance and response reports were used for triangulation and synthesis of findings on selected indicators. It is worthwhile to highlight that the design and samples of each survey differ, and therefore direct comparisons and interpretation of findings needs to be done with great caution.

Data from SPA, SARA and CmONC were used to gain insights on selected indicators of service delivery readiness, assessed by health facility type (such as for hospitals and health centres), during 2008-2018. These indicators include proportion of health facilities offering key MNH services such as: delivery, caesarean section, emergency transport, blood transfusion, and safe abortion, as well as functioning of BEmONC and CEmONC services, and stockouts of uterotonics (parental), neonatal bag and mask and magnesium sulphate. Data for ANC with contents indicator were available in three rounds of SPA and SARA surveys, and therefore, availability of ANC trained staff, blood pressure apparatus, hemoglobin test, urine dipstick/protein, iron tablets (single or with folic acid) and tetanus vaccine, were assessed to gain sights into readiness of health facilities for antenatal care from during 2014-2018.

## E. Measures of inequality within Ethiopia

The national coverage hides the inequalities among the regions within Ethiopia. We used different approaches to visualize and measure geographic and socioeconomic inequalities within Ethiopia. In addition to equiplot used to visualize patterns of coverage of key MNH indicators, absolute difference (highest vs. lowest performing regions, richest vs. poorest, and urban vs. rural in a given year) and average annual rates of change were used to assess changes in coverage, mortality and fertility during 2000-2020.

## F. In-depth literature and document review

In addition to mapping potential data sources, review of literature was used for strategic gathering and analysis of relevant surveys data related to the program outputs, coverage, quality and impact on MNH outcomes, as well as document reviews, and to understand the role of contextual-level drivers and policy levers in achieving these impacts. Extensive reviews of existing articles, reports and documents on national

---

[7] Arroyave L, Saad GE, Victora CG, Barros AJD. A new content-qualified antenatal care coverage indicator: Development and validation of a score using national health surveys in low- and middle-income countries. J Glob Health. 2021 Jan 30;11:04008.

policies and programs and health care financing, released since 1990s, was conducted to gain insights on potential factors that might have been related to the decline in maternal and neonatal mortality.

A literature review was conducted to gather evidence from research studies on changes in coverage and equity of these interventions, and how this might have impacted mortality where available. The review also aimed to gather information from grey literature and peer-reviewed articles on changes in health policies and systems, programs and service outputs, and contextual factors considered to have influenced coverage and equity of those key interventions and their impact on NMR and MMR.

A document review was conducted, involving a retrospective desk review of grey literature and formal documents at the national and subnational level. These were analysed using a narrative synthesis approach to identify information from each document that relates to policy formulation and health system levers (governance, resources/organization payment, financing, regulation, information and communication), health service outputs (access, readiness, quality and integration) as well as program contents and implementation (including facility- and community-based programs and the new interventions introduced therein). The timelines, scale and/or intensity were documented for each component, as well as for identified contextual factors both at national and subnational level. These further used to inform the key informant interview guides, with questions and probes adapted by the implementing team at the state-level for each type of informant.

Furthermore, annual review reports were retrieved and used to collate data on health system inputs and output indicators, while other review of documents such as on maternal death surveillance and response reports were used for triangulation and synthesis of findings. In summary, outputs of the review exercise were used to triangulate and provide a narrative and visual summary of policies, with data synthesized using a policy and program timeline tool, as well as help guide the topic guide development for key informant interviews.

## G. Synthesis

In addition to an inception workshop, which was conducted to gain insights on data sources and local contexts, a national validation and synthesis meeting involving decision makers, program implementers, and other state stakeholders was held to facilitate the interpreted of findings on the country's progress in reducing NMR and MMR and describe the key characteristics of the factors influencing recorded progress. During validation workshop, panel discussions were held to further assess and synthesize results how key policies, programs and contextual factors characterize to country's success on MNH, based on the analyses of program and policy document and secondary quantitative data, as a means of strengthening conclusions the key characteristics of Ethiopia's success over the two decades.

Following these discussions and further synthesis of findings, cross-cutting conclusions were developed to characterize Ethiopia's success in NMR and MMR reduction. In addition, key findings and insights were identified and developed to inform policies, guide strategic planning and support the design and implementation of programs.

## H. More Results

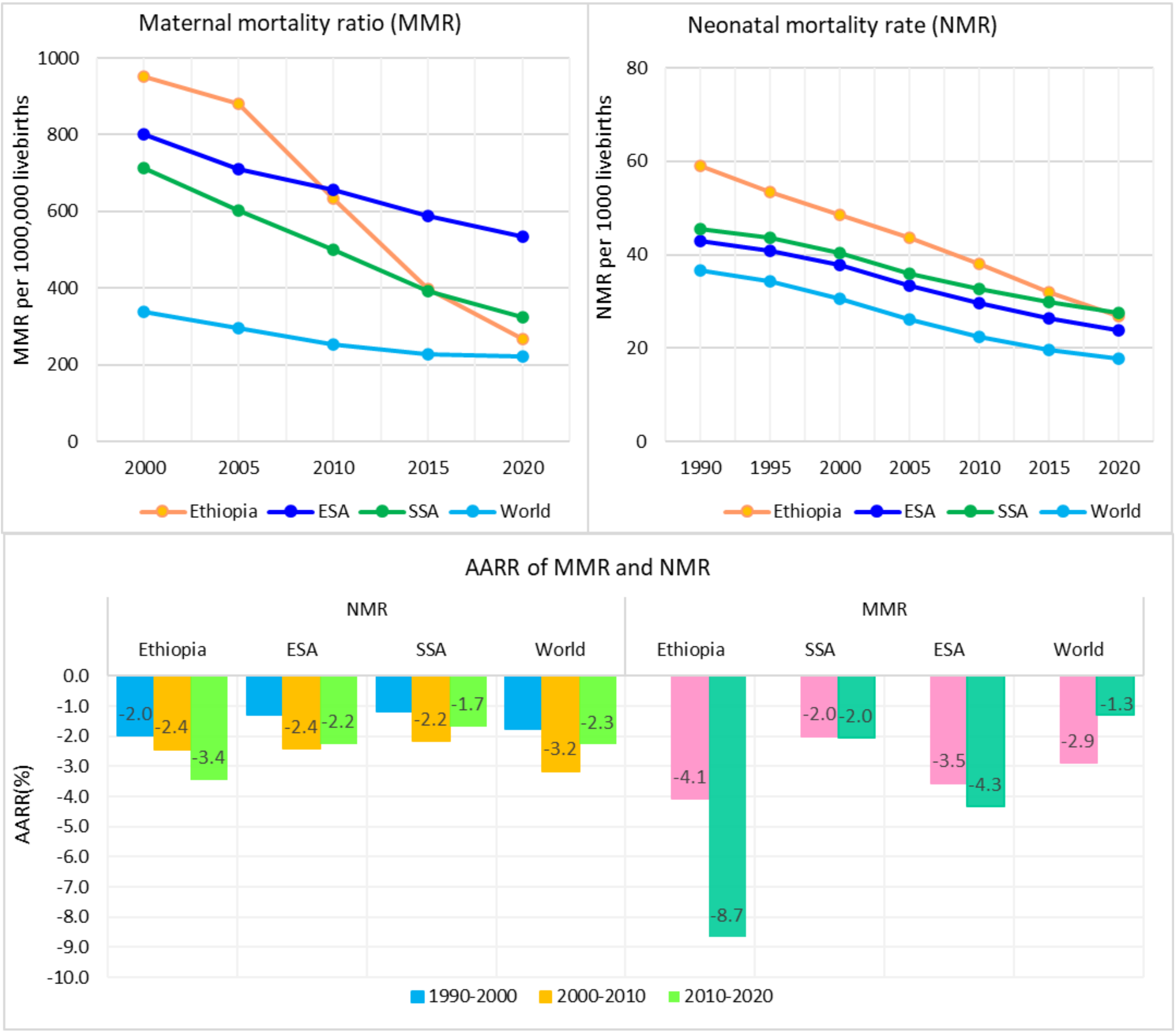


*Appendix Figure 1. Maternal and neonatal mortality in Ethiopia, compared to sub-Saharan Africa (SSA), eastern and southern Africa (ESA) and the world, 1990-2020 (MMR=maternal mortality rate; NMR=neonatal mortality rate).*

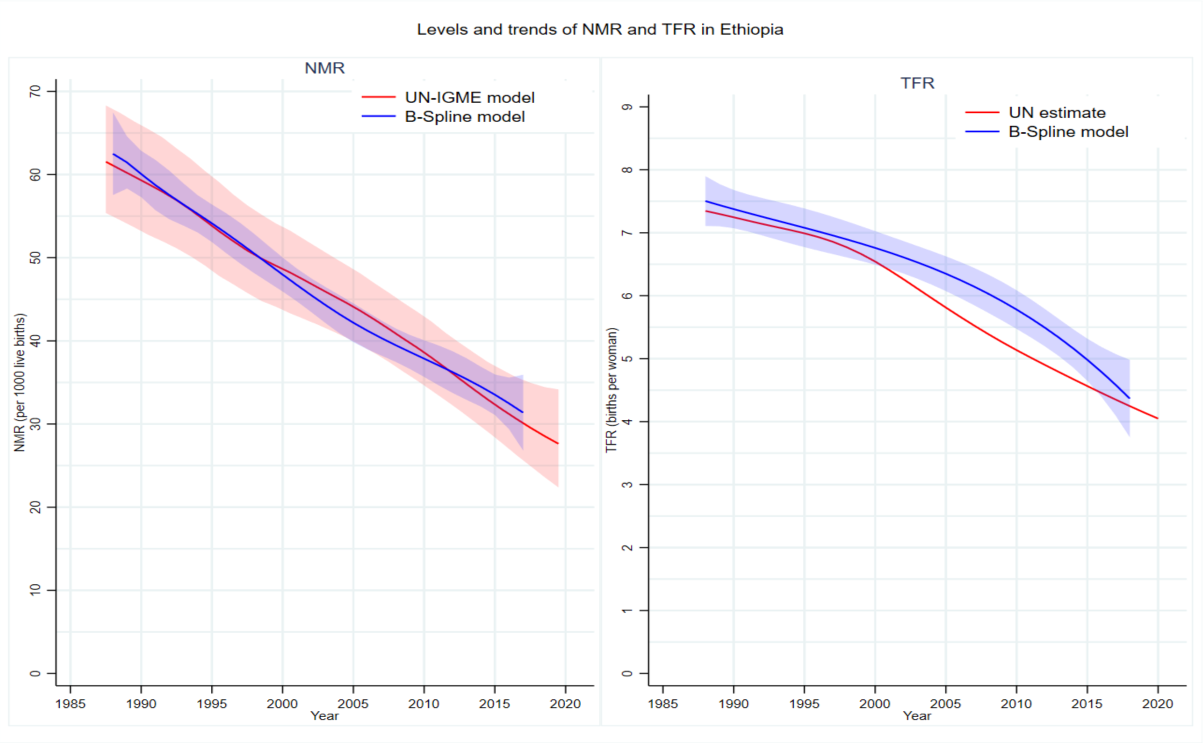


*Appendix Figure 2. NMR and TFR estimates from B-Spline models, plotted against UN-IGME and UN estimates, for triangulation.*

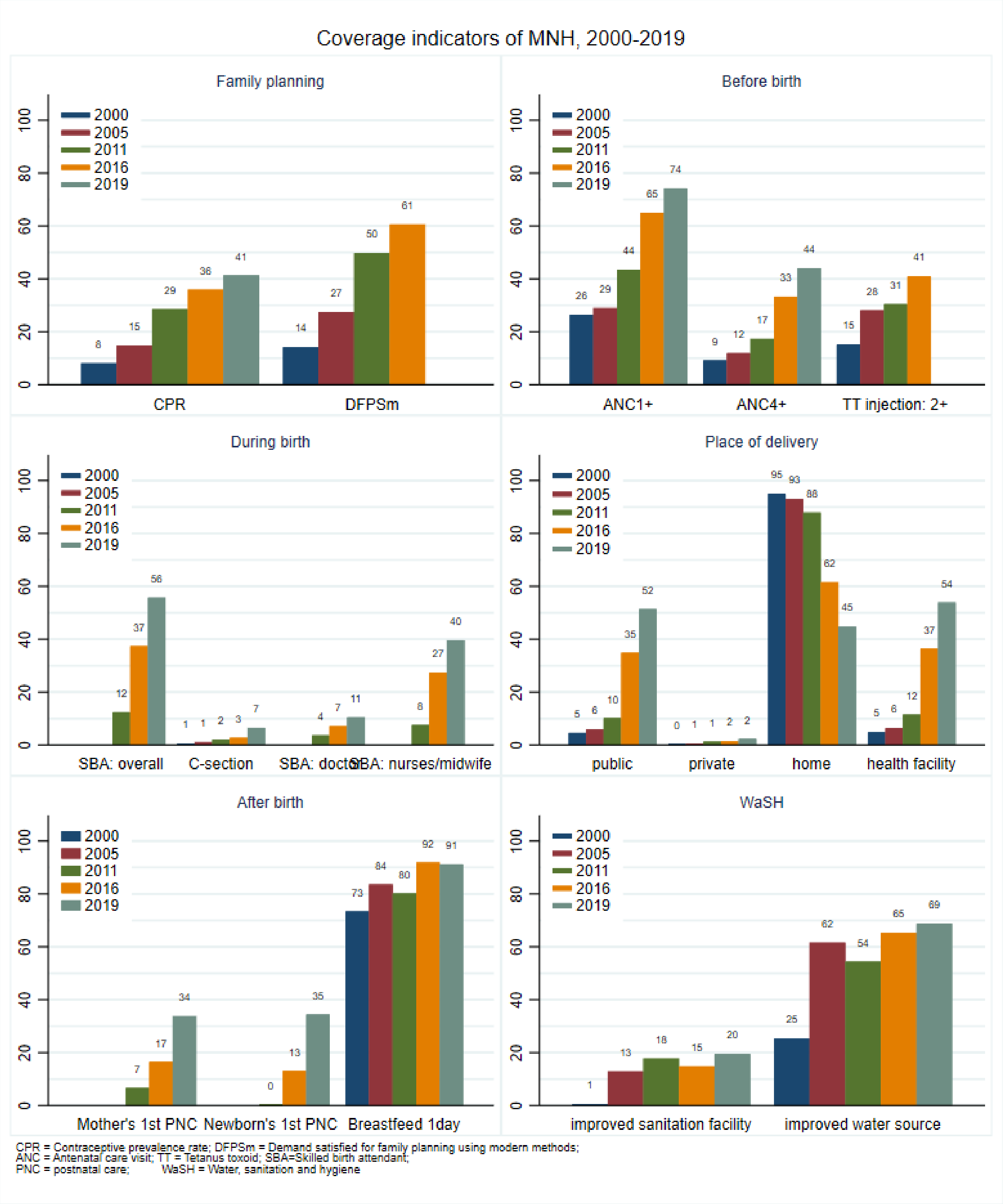


Appendix Figure 3. Key MNH indicators, 2000-2019.

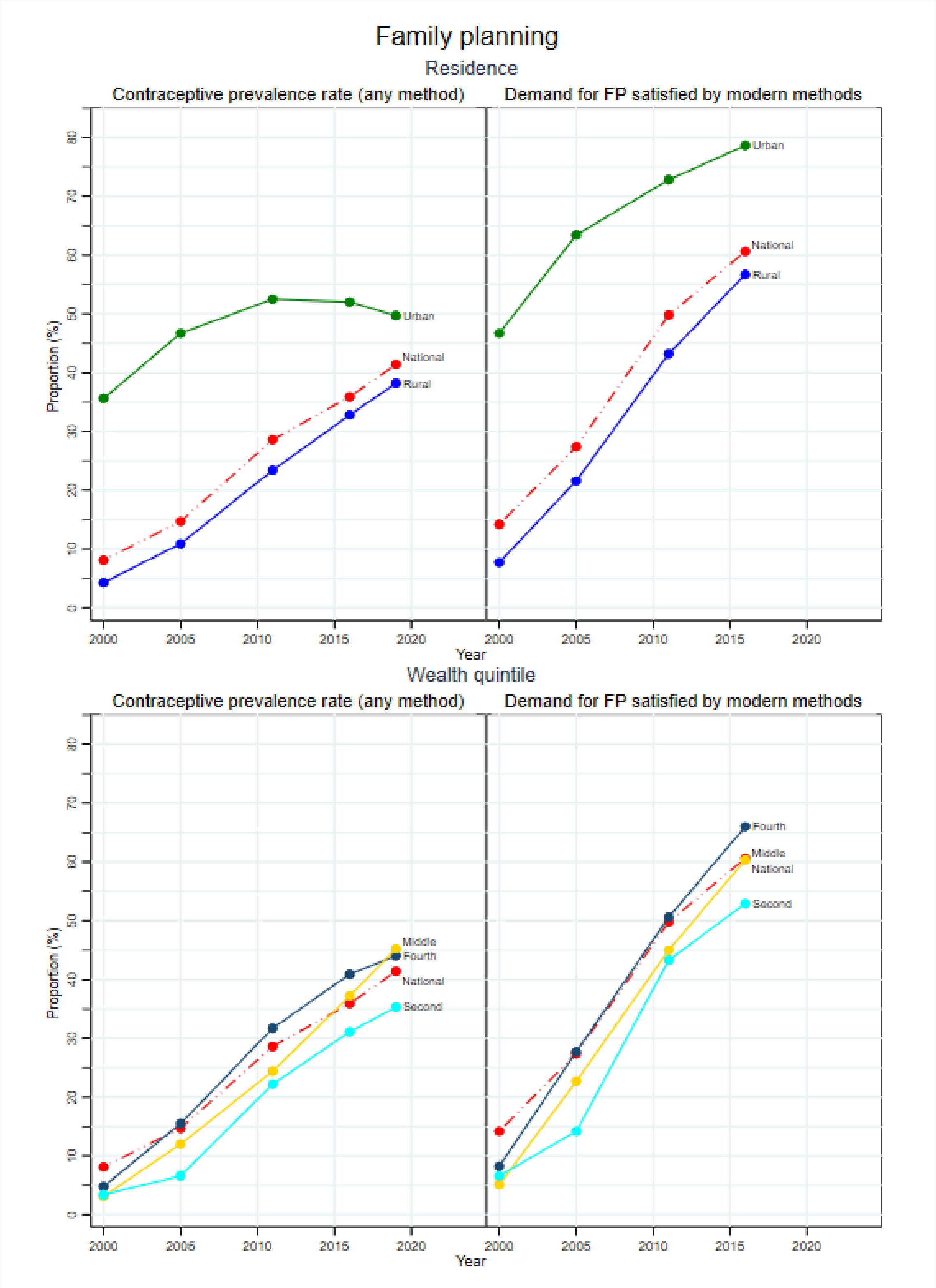


*Appendix Figure 4. Trends in any contraceptive method and demand for family planning satisfied using modern methods, 2000-2019, disaggregated by area of residence and wealth quintile (CPR=contraceptive prevalence rate).*

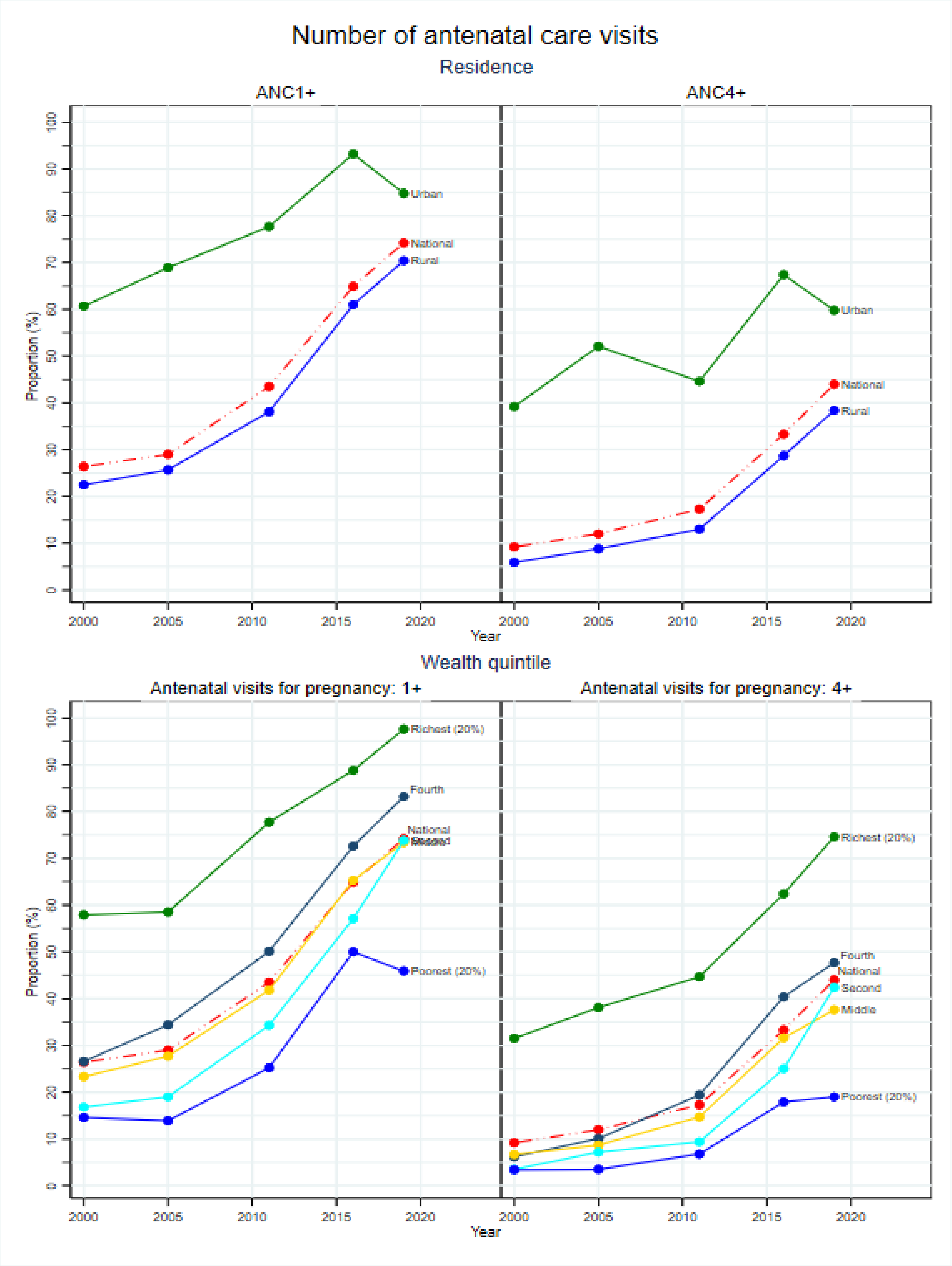


*Appendix Figure 5. Trends of antenatal care visit in Ethiopia, 2000-2019, disaggregated by area of residence and wealth quintile.*

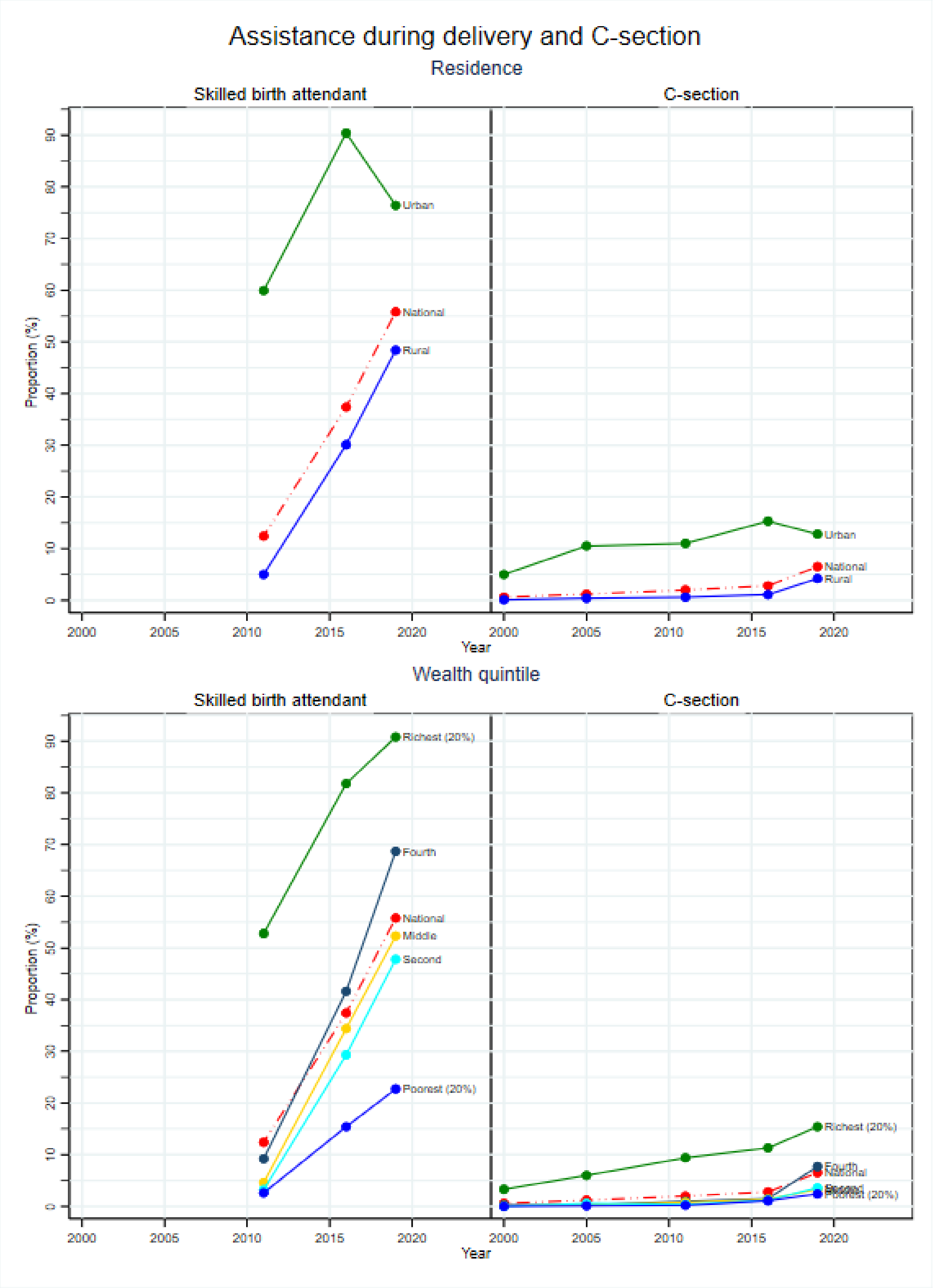


*Appendix Figure 6. Trends of delivery assisted by skilled provider and caesarean section, 2000-2019, disaggregated by area of residence and wealth quintile.*

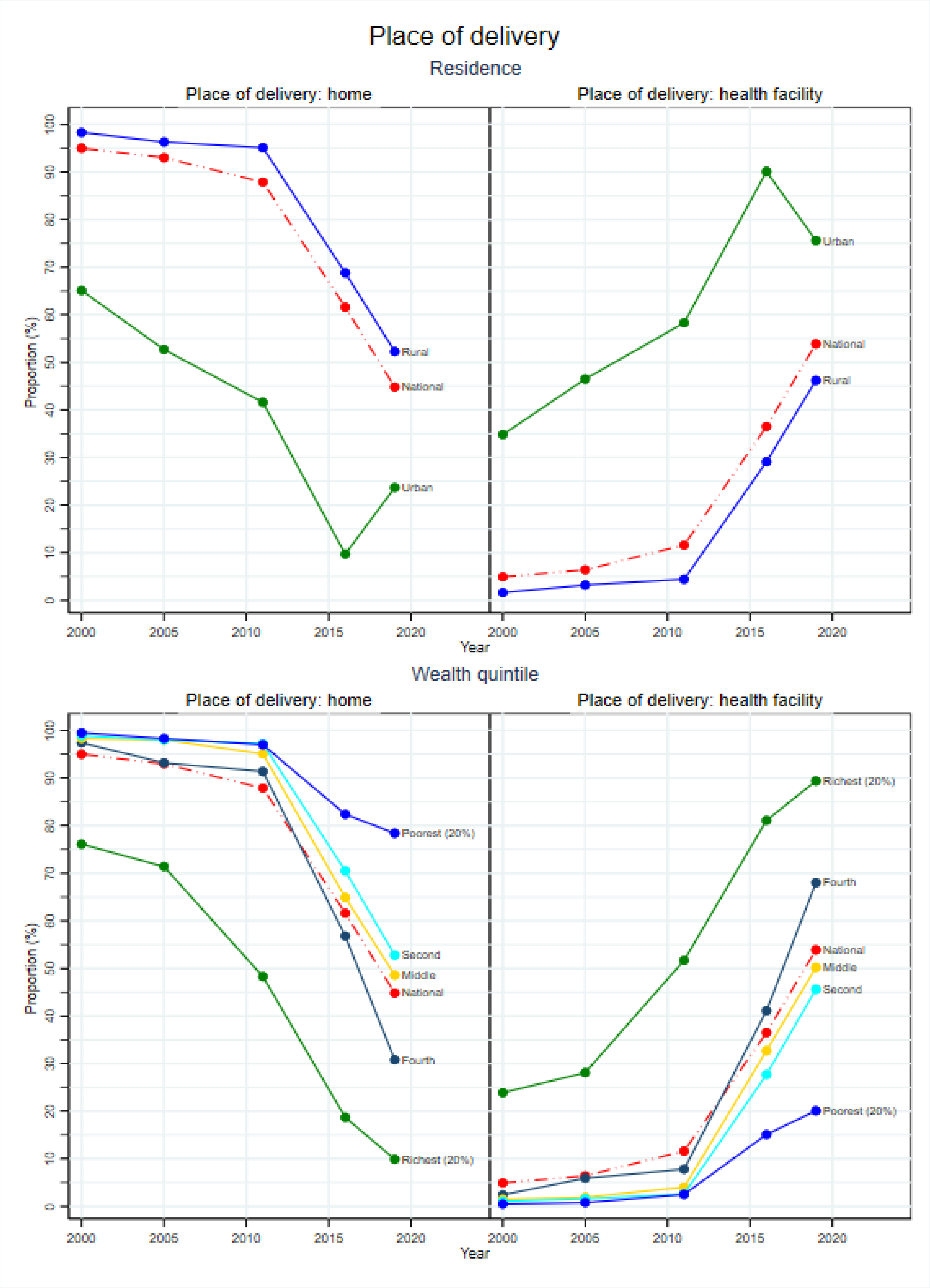


*Appendix Figure 7. Trends of births by place of delivery, 2000-2019, disaggregated by area of residence and wealth quintile.*

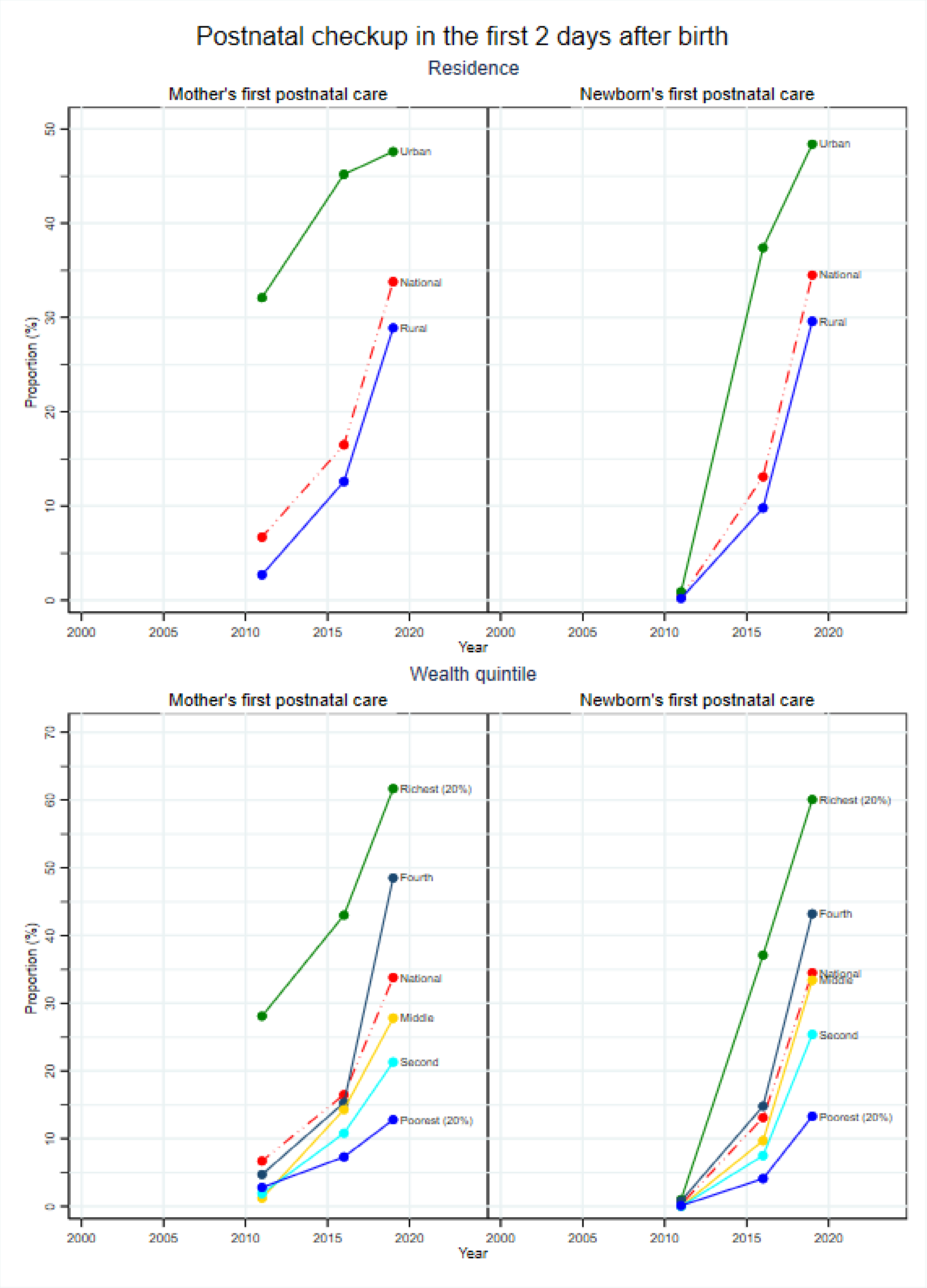


*Appendix Figure 8. Trends of postnatal care for mothers and new-borns, 2000-2019, disaggregated by area of residence and wealth quintile.*

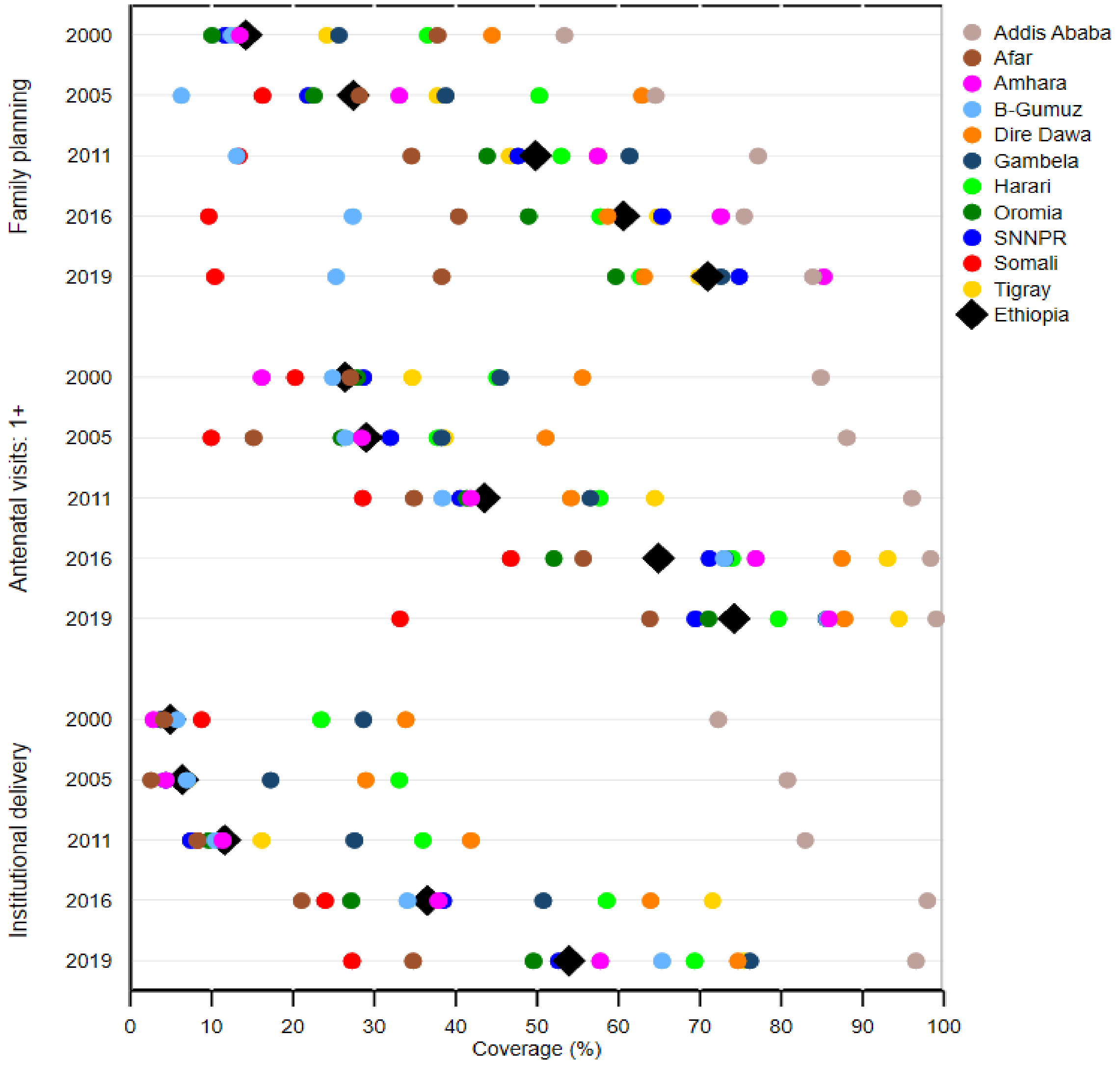


*Appendix Figure 9. Geographical disparities in key MNH indicators during 2000-2019. Note: Family planning presents coverage data for demand satisfied with modern methods, and the 2019 data point for family planning is a linear extrapolation of data from four previous rounds of DHS surveys.*

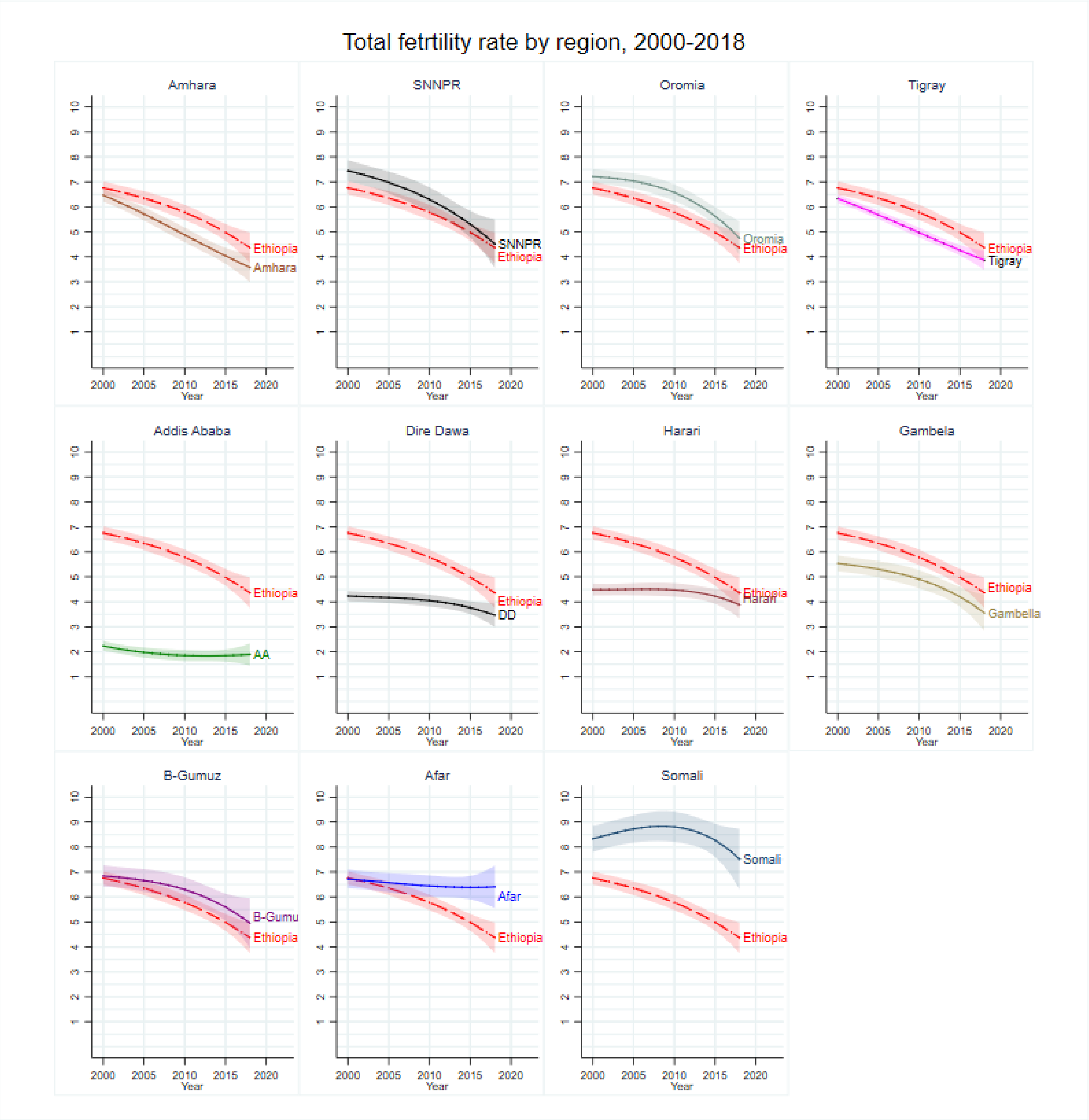


*Appendix Figure 30. Total Fertility rate of Ethiopia, disaggregated by socioeconomic and geographic characteristics, 2000-2019.*

**Appendix Table 1.** Comparison of neonatal mortality rate (NMR), and percentage change and average annual rate of reduction of NMR, by region, 2000 and 2017.

| Region | NMR point estimate[1] | | | Overall change in NMR (%)[2] | | | Average annual rate of reduction (ARR) in NMR (%) | | | | | | | | |
|---|---|---|---|---|---|---|---|---|---|---|---|---|---|---|---|
| | | | | | | | 2000-2010 | | | 2010-2017 | | | 2000-2017 | | |
| | 2000 | 2010 | 2017 | 2000-2010 | 2010-2017 | 2000-2017 | L-UI | ARR PE | U-UI | L-UI | ARR PE | U-UI | L-UI | ARR PE | U-UI |
| Addis Ababa | 25.1 | 16.5 | 17.7 | -34.5 | 7.7 | -29.5 | -5.3 | -4.2 | -3.5 | -1.8 | 1.1 | 2.1 | -4.2 | -2.1 | -0.8 |
| Afar | 41.0 | 34.7 | 26.5 | -15.5 | -23.4 | -35.3 | -2.0 | -1.7 | -1.4 | -4.9 | -3.8 | -1.2 | -4.0 | -2.6 | -1.6 |
| Amhara | 55.2 | 46.2 | 36.8 | -16.3 | -20.3 | -33.3 | -2.0 | -1.8 | -1.6 | -3.5 | -3.2 | -1.3 | -3.2 | -2.4 | -1.7 |
| B-Gumuz | 56.7 | 47.0 | 44.7 | -17.0 | -5.1 | -21.2 | -2.2 | -1.9 | -1.6 | -2.0 | -0.7 | 0.5 | -2.5 | -1.4 | -0.6 |
| Dire Dawa | 38.0 | 32.0 | 32.2 | -15.9 | 0.6 | -15.4 | -2.0 | -1.7 | -1.5 | -1.2 | 0.1 | 1.0 | -1.9 | -1.0 | -0.3 |
| Gambela | 48.0 | 36.3 | 31.4 | -24.5 | -13.5 | -34.6 | -3.1 | -2.8 | -2.5 | -2.9 | -2.1 | -0.4 | -3.5 | -2.5 | -1.7 |
| Harari | 45.2 | 31.8 | 38.9 | -29.7 | 22.5 | -13.9 | -4.1 | -3.5 | -3.0 | 0.9 | 2.9 | 2.8 | -1.9 | -0.9 | -0.1 |
| Oromia | 48.8 | 37.0 | 34.4 | -24.2 | -7.0 | -29.5 | -3.0 | -2.8 | -2.6 | -1.4 | -1.0 | -0.1 | -2.6 | -2.1 | -1.6 |
| SNNPR | 41.1 | 33.0 | 21.3 | -19.6 | -35.5 | **-48.1** | -2.4 | -2.2 | -2.0 | -5.9 | -6.3 | -3.2 | -4.9 | -3.9 | -3.1 |
| Somali | 42.1 | 36.6 | 49.8 | -13.1 | 35.9 | 18.1 | -1.7 | -1.4 | -1.2 | 2.4 | 4.4 | 3.6 | 0.4 | 1.0 | 1.4 |
| Tigray | 51.4 | 32.9 | 27.2 | -36.0 | -17.4 | **-47.1** | -5.0 | -4.5 | -4.0 | -3.9 | -2.7 | -0.6 | -5.2 | -3.7 | -2.7 |
| Ethiopia[3] | 48.2 | 37.7 | 32.0 | -21.7 | -15.3 | -33.7 | -2.6 | -2.4 | -2.3 | -2.4 | -2.4 | -1.0 | -2.9 | -2.4 | -2.0 |

Positive numbers in the last eight columns indicate increase in neonatal mortality rate (NMR), rather than reduction.

[1]NMR (neonatal mortality rate per 1,000 live births) – estimates have been rounded to the nearest 1.

[2]Overall change for the two periods represent data from 2000 to 2010 and 2010 to 2017.

3Neonatal mortality rate estimate for Ethiopia was generated using the same model that was used for subnational estimation for comparison purposes. Our estimation of mortality was triangulated with UN estimate, and summary measures of progress at national level using both approaches are nearly the same.

**Note:** NMR has increased in Harari and Somali while it has shown little or no change in Addis Ababa, Benishangul Gumuz and Dire Dawa during 2010-2017.

PE, L-UI and U-UI refers to ARR point estimate, and lower and upper uncertainty interval for point estimate of neonatal mortality rate, respectively.

**Appendix Table 2.** Comparison of total fertility rate (TFR), and percentage change and average annual rate of reduction of TFR, by region, 2000 and 2018.

| Region | MMR point estimate[1] | | | Overall change in NMR (%)[2] | | | Average annual rate of reduction (ARR) in NMR (%) | | | | | | | | |
|---|---|---|---|---|---|---|---|---|---|---|---|---|---|---|---|
| | | | | | | | 2000-2010 | | | 2010-2018 | | | 2000-2018 | | |
| | 2000 | 2010 | 2018 | 2000-2010 | 2010-2018 | 2000-2018 | L-UI | ARR PE | U-UI | L-UI | ARR PE | U-UI | L-UI | ARR PE | U-UI |
| Addis Ababa | 2.2 | 1.9 | 1.9 | -17.2 | 2.1 | -15.5 | -2.3 | **-1.9** | -1.6 | -1.5 | **0.3** | 1.5 | -1.9 | **-0.9** | -0.2 |
| Afar | 6.7 | 6.4 | 6.4 | -4.1 | -0.6 | -4.7 | -0.5 | **-0.4** | -0.3 | -1.0 | **-0.1** | 0.7 | -0.7 | **-0.3** | 0.1 |
| Amhara | 6.5 | 4.9 | 3.6 | -24.5 | -26.7 | -44.7 | -3.0 | **-2.8** | -2.6 | -5.3 | **-3.9** | -2.7 | -4.1 | **-3.3** | -2.7 |
| B-Gumuz | 6.8 | 6.3 | 5.0 | -8.0 | -21.3 | -27.6 | -1.0 | **-0.8** | -0.7 | -4.8 | **-3.0** | -1.6 | -2.7 | **-1.8** | -1.1 |
| Dire Dawa | 4.2 | 4.1 | 3.5 | -4.2 | -14.5 | -18.1 | -0.5 | **-0.4** | -0.3 | -3.0 | **-2.0** | -1.1 | -1.7 | **-1.1** | -0.7 |
| Gambela | 5.5 | 4.9 | 3.6 | -11.5 | -27.4 | -35.7 | -1.4 | **-1.2** | -1.1 | -5.9 | **-4.0** | -2.6 | -3.4 | **-2.5** | -1.7 |
| Harari | 4.5 | 4.5 | 3.9 | -0.3 | -13.3 | -13.6 | -0.1 | **0.0** | 0.0 | -2.9 | **-1.8** | -0.9 | -1.4 | **-0.8** | -0.4 |
| Oromia | 7.2 | 6.6 | 4.8 | -9.0 | -27.7 | -34.2 | -1.1 | **-0.9** | -0.8 | -5.3 | **-4.1** | -3.0 | -2.9 | **-2.3** | -1.8 |
| SNNPR | 7.4 | 6.3 | 4.5 | -15.5 | -28.1 | -39.2 | -1.9 | **-1.7** | -1.5 | -6.2 | **-4.1** | -2.6 | -3.8 | **-2.8** | -2.0 |
| Somali | 8.3 | 8.8 | 7.5 | 5.8 | -14.7 | -9.7 | 0.5 | **0.6** | 0.6 | -3.3 | **-2.0** | -0.9 | -1.2 | **-0.6** | -0.1 |
| Tigray | 6.3 | 5.0 | 3.9 | -21.5 | -22.3 | -39.0 | -2.5 | **-2.4** | -2.3 | -4.0 | **-3.1** | -2.4 | -3.2 | **-2.7** | -2.4 |
| Ethiopia | 6.8 | 5.8 | 4.4 | -14.5 | -24.5 | -35.4 | -1.7 | **-1.6** | -1.4 | -4.7 | **-3.5** | -2.5 | -3.0 | **-2.4** | -1.9 |

Positive numbers in the last twelve columns indicate increase in total fertility rate (TFR), rather than reduction.
[1]TFR (number of children per woman) – estimates have been rounded to the nearest 1.
[2]Overall change for the period (2000-2018), and two periods represent data from 2000 to 2010 and 2010 to 2018.
[3]TFR estimate for Ethiopia was generated using the same model that was used for subnational estimation for comparison purposes. Our estimation of fertility was triangulated with UN estimates, and summary measures of progress at national level using both approaches are nearly the same.
**Note:** TFR has shown little or no change in Addis Ababa, Afar and Harari during 2010-2018.
PE, L-UI and U-UI refers to point estimate, and lower and upper uncertainty interval for point estimate of total fertility rate, respectively.

Appendix Table 3: Coverage of selected MNH indicators and changes overtime, 2000-2019.

| | | Coverage (%)[1] | | | Overall change in coverage (%)[2] | | | Average annual rate of reduction (ARR) in coverage (%) | | |
|---|---|---|---|---|---|---|---|---|---|---|
| MNH indicator | Region | 2000 | 2011 | 2019 | 2000-2011 | 2011-2018 | 2000-2019 | 2000-2011 | 2011-2018 | 2000-2019 |
| Family planning[3] | Addis Ababa | 53.3 | 77.1 | 83.8 | 44.7 | 8.7 | 57.3 | 3.4 | 1.0 | 2.4 |
| | Afar | 37.7 | 34.5 | 38.2 | -8.5 | 10.8 | 1.4 | -0.8 | 1.3 | 0.1 |
| | Amhara | 13.4 | 57.4 | 85.2 | 328.4 | 48.4 | 535.5 | 13.2 | 4.9 | 9.7 |
| | B-Gumuz | 12.4 | 13.0 | 25.2 | 4.8 | 94.0 | 103.3 | 0.4 | 8.3 | 3.7 |
| | Dire Dawa | 44.4 | 57.4 | 63.1 | 29.3 | 9.9 | 42.1 | 2.3 | 1.2 | 1.8 |
| | Gambela | 25.6 | 61.3 | 72.6 | 139.5 | 18.4 | 183.4 | 7.9 | 2.1 | 5.5 |
| | Harari | 36.5 | 52.9 | 62.6 | 44.9 | 18.4 | 71.6 | 3.4 | 2.1 | 2.8 |
| | Oromia | 10.0 | 43.8 | 59.6 | 338.0 | 36.1 | 496.1 | 13.4 | 3.9 | 9.4 |
| | SNNPR | 11.6 | 47.6 | 74.8 | 310.3 | 57.1 | 544.7 | 12.8 | 5.6 | 9.8 |
| | Somali | 13.2 | 13.3 | 10.4 | 0.8 | -22.2 | -21.6 | 0.1 | -3.1 | -1.3 |
| | Tigray | 24.1 | 46.6 | 69.8 | 93.4 | 49.9 | 189.8 | 6.0 | 5.1 | 5.6 |
| | Ethiopia | 14.2 | 49.8 | 71.0 | 250.7 | 42.5 | 399.7 | 11.4 | 4.4 | 8.5 |
| | Abs. diff* | 43.3 | 64.1 | 74.8 | | | | 3.6 | 1.9 | 2.9 |
| Antenatal visit: 1+ | Addis Ababa | 84.8 | 96.0 | 99.0 | 13.2 | 3.1 | 16.7 | 1.1 | 0.4 | 0.8 |
| | Afar | 27.0 | 34.8 | 63.8 | 28.9 | 83.3 | 136.3 | 2.3 | 7.6 | 4.5 |
| | Amhara | 16.1 | 41.8 | 85.8 | 159.6 | 105.3 | 432.9 | 8.7 | 9.0 | 8.8 |
| | B-Gumuz | 24.8 | 38.3 | 85.6 | 54.4 | 123.5 | 245.2 | 4.0 | 10.1 | 6.5 |
| | Dire Dawa | 55.5 | 54.1 | 87.7 | -2.5 | 62.1 | 58.0 | -0.2 | 6.0 | 2.4 |
| | Gambela | 45.4 | 56.5 | 85.5 | 24.4 | 51.3 | 88.3 | 2.0 | 5.2 | 3.3 |
| | Harari | 45.0 | 57.6 | 79.6 | 28.0 | 38.2 | 76.9 | 2.2 | 4.0 | 3.0 |
| | Oromia | 27.8 | 41.3 | 71.0 | 48.6 | 71.9 | 155.4 | 3.6 | 6.8 | 4.9 |
| | SNNPR | 28.6 | 40.5 | 69.4 | 41.6 | 71.4 | 142.7 | 3.2 | 6.7 | 4.7 |
| | Somali | 20.2 | 28.5 | 33.1 | 41.1 | 16.1 | 63.9 | 3.1 | 1.9 | 2.6 |
| | Tigray | 34.6 | 64.4 | 94.4 | 86.1 | 46.6 | 172.8 | 5.6 | 4.8 | 5.3 |
| | Ethiopia | 26.4 | 43.5 | 74.2 | 64.8 | 70.6 | 181.1 | 4.5 | 6.7 | 5.4 |
| | Abs. diff* | 68.7 | 67.5 | 65.9 | | | | -0.2 | -0.3 | -0.2 |
| Institution al delivery | Addis Ababa | 72.2 | 82.9 | 96.5 | 14.8 | 16.4 | 33.7 | 1.3 | 1.9 | 1.5 |
| | Afar | 4.1 | 8.2 | 34.7 | 100.0 | 323.2 | 746.3 | 6.3 | 18.0 | 11.2 |
| | Amhara | 2.8 | 11.3 | 57.7 | 303.6 | 410.6 | 1960.7 | 12.7 | 20.4 | 15.9 |
| | B-Gumuz | 5.7 | 10.4 | 65.3 | 82.5 | 527.9 | 1045.6 | 5.5 | 23.0 | 12.8 |
| | Dire Dawa | 33.8 | 41.8 | 74.6 | 23.7 | 78.5 | 120.7 | 1.9 | 7.2 | 4.2 |
| | Gambela | 28.6 | 27.5 | 76.1 | -3.8 | 176.7 | 166.1 | -0.4 | 12.7 | 5.2 |
| | Harari | 23.4 | 35.9 | 69.3 | 53.4 | 93.0 | 196.2 | 3.9 | 8.2 | 5.7 |
| | Oromia | 4 | 9.6 | 49.5 | 140.0 | 415.6 | 1137.5 | 8.0 | 20.5 | 13.2 |
| | SNNPR | 3.7 | 7.4 | 52.6 | 100.0 | 610.8 | 1321.6 | 6.3 | 24.5 | 14.0 |
| | Somali | 8.7 | 8.2 | 27.2 | -5.7 | 231.7 | 212.6 | -0.5 | 15.0 | 6.0 |
| | Tigray | 3.6 | 16.1 | 75.4 | 347.2 | 368.3 | 1994.4 | 13.6 | 19.3 | 16.0 |
| | Ethiopia | 4.9 | 11.6 | 53.9 | 136.7 | 364.7 | 1000.0 | 7.8 | 19.2 | 12.6 |
| | Abs. diff* | 69.4 | 75.5 | 69.3 | | | | 0.8 | -1.1 | 0.0 |

[1]Data represents two years period preceding each survey.
[2]The two periods represent data from 2000 to 2011 and 2011 to 2019 for comparison, and the overall is for 2000-2019.
[3]Family planning refers to demand satisfied by modern methods, with the 2019 data obtained from a linear extrapolation of four previous DHS round. *Abs. diff. refers to absolute difference between regions with two extreme coverage (minimum and maximum coverage).

Appendix Table 4: Trends in density of health professionals (per 10,000 people) at regional level, 2000-2020

| **Region** | **General practitioners + specialists** | | | **Health officers** | | | **Nurses** | | | **Midwives** | | |
|---|---|---|---|---|---|---|---|---|---|---|---|---|
| | **2000** | **2010** | **2020** | **2000** | **2010** | **2020** | **2000** | **2010** | **2020** | **2000** | **2010** | **2020** |
| Tigray | 0.2 | 0.2 | 1.6 | 0.1 | 0.7 | 1.9 | 2.4 | 5.8 | 11.3 | 0.4 | 0.5 | 2.7 |
| Afar | 0.1 | 0.1 | 0.4 | 0.1 | 0.3 | 1.2 | 1.9 | 3.3 | 5.5 | 0.2 | 0.1 | 1.3 |
| Amhara | 0.2 | 0.1 | 1.1 | 0.1 | 0.4 | 1.5 | 1.0 | 2.6 | 5.5 | 0.1 | 0.2 | 2.4 |
| Oromia | 0.1 | 0.1 | 0.6 | 0.1 | 0.4 | 0.9 | 0.8 | 3.4 | 4.8 | 0.1 | 0.3 | 1.2 |
| Somali | 0.1 | 0.2 | 1.1 | 0.0 | 0.2 | 1.1 | 0.9 | 3.5 | 5.1 | 0.1 | 0.5 | 2.9 |
| B-Gumuz | 0.4 | 0.3 | 0.9 | 0.2 | 1.1 | 1.9 | 3.6 | 7.0 | 13.9 | 0.4 | 0.3 | 4.9 |
| SNNPR | 0.1 | 0.1 | 0.9 | 0.0 | 0.4 | 2.1 | 0.9 | 2.4 | 7.3 | 0.1 | 0.3 | 2.1 |
| Gambela | 0.4 | 0.4 | 1.4 | 0.4 | 1.4 | 2.9 | 6.1 | 8.7 | 23.1 | 0.7 | 0.1 | 1.2 |
| Harari | 3.2 | 2.0 | 2.9 | 0.1 | 2.1 | 2.0 | 9.7 | 13.0 | 15.4 | 0.9 | 2.2 | 4.1 |
| Dire Dawa | 1.2 | 0.9 | 2.7 | 0.1 | 1.3 | 1.3 | 3.8 | 9.4 | 8.9 | 0.3 | 1.3 | 2.1 |
| Addis | 2.1 | 1.5 | 8.2 | 0.1 | 1.0 | 7.7 | 5.0 | 13.6 | 23.7 | 0.5 | 0.7 | 4.0 |
| **Ethiopia** | **0.2** | **0.2** | **1.2** | **0.1** | **0.5** | **1.6** | **1.2** | **3.6** | **6.8** | **0.1** | **0.3** | **2.0** |

Note: all density values are rounded to one decimal place (i.e., 0.0 values do not necessarily mean zero density).

Appendix Table 5. Selected readiness indicators for antenatal care from facility assessments, 2014-2018.

| | **SPA 2014** | **SARA 2016** | **SARA 2018** |
|---|---|---|---|
| Number of facilities | 1165 | 698 | 764 |
| Antenatal care offered (%) | 87 | 80 | 78 |
| At least one ANC trained staff (%) | 54 | 44 | 31 |
| Blood pressure apparatus present (%) | 71 | 69 | 54 |
| Hemoglobin test available (%) | 26 | 8 | 6 |
| Urine dipstick, protein (%) | 52 | 15 | 15 |
| Iron tablets (single or with folic acid, %) | 52 | 66 | 38 |
| Tetanus toxoid vaccine (%) | 42 | 60 | 39 |

Appendix Table 6: Selected indicators of service delivery readiness for hospitals and health centres in facility assessments during 2008-2018.

| | **EmONC 2008** | **EmONC 2016** | **SPA 2014** | **SARA 2016** | **SARA 2018** |
|---|---|---|---|---|---|
| Number of facilities | | | | | |
| ... Hospitals | 112 | 316 | 214 | 210 | 303 |
| ... Health centers | 685 | 3488 | 292 | 165 | 164 |
| Offers delivery services (%) | 94 | 100 | 100 | 69 | 76 |
| Uterotonics (parenteral): no stockouts in last 3 months (%) | | | | | |
| ... Hospitals | 75 | 95 | 88 | 98 | 99 |
| ... Health centers | 43 | 70 | 75 | 84 | 93 |
| Magnesium sulphate: no stockout in last 3 months (%) | | | | | |
| ... Hospitals | 97 | 72 | 69 | 78 | 88 |
| ... Health centers | 97 | 45 | 22 | 37 | 69 |
| Neonatal bag and mask, no stockouts in last 3 months (%) | | | | | |
| ... Hospitals | | 86 | 98 | 88 | 88 |
| ... Health centers | 33 | 72 | 81 | 77 | 68 |
| Emergency transport | | | | | |
| ... Hospitals | 86 | 90 | 92 | 99 | 92 |
| ... Health centers | 41 | 13 | 91 | 85 | 76 |
| Offer CS (%) | 78 | 80 | 83 | 86 | 91 |
| Offers blood transfusion | 64 | 65 | 78 | 82 | 82 |
| BEmONC (fully functioning) | | | | | |
| ... Hospitals | 65 | 59 | | | |
| ... Health centers | 1 | 5 | | | |
| CEmONC (fully functioning) | | | | | |
| ... Hospitals | 51 | 45 | | 73 | 75 |
| Offers safe abortion services (%) | 36 | 42 | | | |
